# Genetic modification of the flavonoid pathway alters growth and reveals flexible responses to enhanced UVB – Role of foliar condensed tannins

**DOI:** 10.1002/pei3.10036

**Published:** 2020-12-28

**Authors:** Paula Thitz, Ann E. Hagerman, Tendry R. Randriamanana, Virpi Virjamo, Minna Kosonen, Mika Lännenpää, Tommi Nyman, Lauri Mehtätalo, Sari Kontunen‐Soppela, Riitta Julkunen‐Tiitto

**Affiliations:** ^1^ Department of Environmental and Biological Sciences University of Eastern Finland Joensuu Finland; ^2^ Department of Chemistry and Biochemistry Miami University Oxford OH USA; ^3^ Department of Ecosystems in the Barents Region Norwegian Institute of Bioeconomy Research Svanvik Norway; ^4^ School of Computing University of Eastern Finland Joensuu Finland; ^5^ Present address: School of Forest Sciences University of Eastern Finland Joensuu Finland; ^6^ Present address: Natural Resources Institute Finland Mikkeli Finland; ^7^ Present address: Biocarelia Research Laboratory Juurikka Finland

**Keywords:** condensed tannins, flavonoids, UV light, RNA interference, oxidative stress, trichomes, photosynthesis, plant development, *Betula*, polyphenols

## Abstract

Accumulation of certain phenolics is a well‐known response of plants to enhanced UVB radiation (280–315 nm), but few experiments have compared the relative importance of different phenolic groups for UVB resilience. To study how an altered phenolic profile affects the responses and resilience of silver birch (*Betula pendula*) to enhanced UVB, we used RNA interference (RNAi) targeting dihydroflavonol reductase (DFR), anthocyanidin synthase (ANS), or anthocyanidin reductase (ANR) to change the accumulation of phenolics. The unmodified control line and RNAi‐modified plants were grown for 51 days under ambient or +32% enhanced UVB dose in a greenhouse. RNAi greatly affected phenolic profile and plant growth. There were no interactive effects of RNAi and UVB on growth or photosynthesis, which indicates that the RNAi and unmodified control plants were equally resilient. UVB enhancement led to an accumulation of foliar flavonoids and condensed tannins, and an increase in the density of stem glands and glandular trichomes on upper leaf surfaces in both the control and RNAi‐modified plants. Our results do not indicate a photoprotective role for condensed tannins. However, decreased growth of high‐flavonoid low‐tannin DFRi and ANRi plants implies that the balance of flavonoids and condensed tannins might be important for normal plant growth.

## 1 INTRODUCTION

Ultraviolet‐B radiation (UVB) has profound biological effects on plants, although it constitutes less than 0.5% of the light energy reaching the Earth's surface (Heijde & Ulm, 2012; Robson, Klem, et al., 2015). The well‐known effects of UVB in plants include the accumulation of phenolic compounds (Holopainen et al., 2018; Li et al., 2010), damage to DNA and photosynthetic machinery (Giordano et al., 2004; Kataria et al., 2014) and increased oxidative pressure (Hideg et al., 2013). The accumulation of phenolics has been linked to the UVB‐specific photoreceptor UVR8 (Brown et al., 2005; Kliebenstein et al., 2002), which upregulates the phenylpropanoid pathway producing flavonoids, anthocyanidins and condensed tannins from smaller phenolic precursors (Quideau et al., 2011). This, together with the early identification of phenylpropanoid pathway genes conferring susceptibility or tolerance to high UVB in *Arabidopsis thaliana* Schur (Bieza & Lois, 2001; Li et al., 1993), provides strong support for the UVB‐protective role of plant phenolics.

**FIGURE 1:**
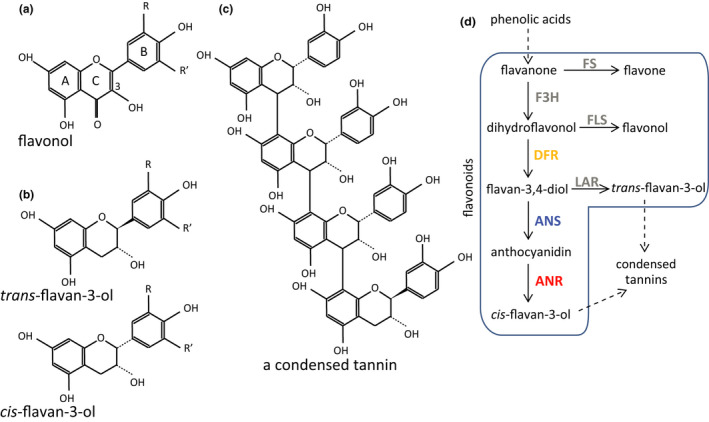
Structures of focal phenolic compounds and simplified flavonoid‐tannin pathway of *Betula pendula*. (a) Flavonol (kaempferol R=R’=H; quercetin R=H, R’=OH; myricetin R=R’=OH), with rings and typically glycosylated C3 labelled. (b) Flavan‐3‐ols with either 2,3‐*cis‐* (epicatechin R=H, R’=OH; epigallocatechin R=R’=OH) or 2,3‐*trans*‐configuration (catechin R=H, R’=OH; gallocatechin R=R’=OH). (c) A simple linear condensed tannin molecule composed of ortho‐dihydroxylated catechin (terminal) and epicatechin (extender) subunits. (d) Flavone synthase, FS; flavanone 3‐dioxygenase, F3H; flavonol synthase, FLS; dihydroflavonol reductase, DFR; leucoanthocyanidin reductase, LAR; anthocyanidin synthase, ANS; and anthocyanidin reductase, ANR. Enzymes restricted by RNA interference are colored

Plant phenolics could protect plants by acting as UVB screens that reduce the amount of incident radiation reaching the mesophyll cells, as has been shown for phenolic acids and flavonoids (Bidel et al., 2007). Alternatively, these compounds could alleviate UVB‐induced oxidative stress (Hernández et al., 2009). Flavonoid compounds with an ortho‐dihydroxylated B‐ring (as in quercetins, Figure 1a) are particularly effective antioxidants in vitro (Williams et al., 2004). Accumulation of phenolics, especially in epidermal tissues exposed to solar radiation or within secretory glandular trichomes, could enhance their effectiveness in UVB protection (Liakoura et al., 1997; Tattini et al., 2004).

Despite the generally accepted view of UVB‐protective capacities of phenolic compounds such as flavonoids (Julkunen‐Tiitto et al., 2005), these properties have only recently been suggested for condensed tannins, which are alternative end products of the UVB‐responsive phenylpropanoid pathway (Close & McArthur, 2002; Mellway & Constabel, 2009). Condensed tannins (also known as proanthocyanidins) are phenolic polymers often associated with antiherbivore functions (Marsh et al., 2020). Condensed tannins are composed of flavan‐3‐ol‐derived subunits (Figure 1b–d) and usually contain *ortho* di‐ or trihydroxylated structures that are good reducing agents (Hagerman et al., 1998; Xie & Dixon, 2005), making them potential players in plant defense against UVB‐related oxidative stress. Accumulation of condensed tannins in poplar is regulated by MYB115 and MYB134 transcription factors, which could act as a part of MBW complex that upregulates biosynthetic pathways leading to the accumulation of both flavonoids and condensed tannins (James et al., 2017). *MYB134*‐ or *MYB115*‐mediated upregulation of the whole flavonoid‐tannin pathway in transgenic *Populus tremula* × *tremuloides* has been linked with increased resistance to oxidative stress (Gourlay & Constabel, 2019). Either the condensed tannins, or the flavonoids that accumulate in these plant lines could contribute to UV resistance (James et al., 2017; Mellway et al., 2009). Therefore, additional studies that directly target the production of condensed tannins are required to elucidate the potential UVB‐protective roles of condensed tannins in deciduous trees.

Besides the consistent UVB‐related accumulation of plant phenolics, UVB causes variable morphological and physiological responses in plants. These responses depend on inherent differences in resistance or tolerance (Ren et al., 2007) and the exposure to UVB and longer wavelengths, including ultraviolet‐A (UVA; 315–400 nm) and photosynthetically active radiation (PAR; 400–700 nm; Krizek, 2004). Plants grown under high UVB often have smaller aboveground biomass or altered architecture, with shorter internodes and increased branching (Li et al., 2010; Robson, Klem, et al., 2015). Meta‐analyses summarizing the effects of UVB on photosynthetic parameters indicate contradictory results (Li et al., 2010; Searles et al., 2001), suggesting that ecologically relevant UVB levels may impair photosynthesis only in sensitive species or non‐acclimated plants (Hunt & McNeil, 1999; Wargent et al., 2015). Recently, UVB doses below plant tolerance limits have been suggested to have regulatory or beneficial effects. By activating plant antioxidant defenses, low UVB levels could improve plant resistance to environmental stressors (Hideg et al., 2013).

In this study, we investigated how an altered phenolic profile affects the responses and resilience of silver birch (*Betula pendula* Roth) to enhanced UVB, and assessed the role of condensed tannins in UVB protection. We did this by constructing genetically modified *B. pendula* lines in which the biosynthetic pathway producing condensed tannins had been partially blocked, and exposing these plants to ambient and enhanced doses of UVB for 51 days in a greenhouse. RNA interference (RNAi) was used to reduce the levels of dihydroflavonol reductase (DFR), anthocyanidin synthase (ANS) or anthocyanidin reductase (ANR; Figure 1d), enzymes whose blockage causes accumulation of flavonoids at the expense of condensed tannins, or alters the structures of condensed tannins in *B. pendula* (Thitz et al., 2020). To decipher whether the different RNAi plants have different capacities to acclimate to stress caused by enhanced UVB, we followed plant growth and leaf chlorophyll content, and measured photosynthesis, evaporation, and photosystem II efficiency during the experiment. We connect these findings to the phenolic composition (low‐molecular weight phenolics and condensed tannins) and morphological variables measured from leaves and stems at the end of the experiment, and discuss our findings in the wider context of chemical defenses in woody plants.

## 2 MATERIALS AND METHODS

### 2.1 Plant material

RNA interference was used to decrease the expression of dihydroflavonol reductase, anthocyanidin synthase or anthocyanidin reductase (Figure 1d; transformed plants hereafter called “DFRi”, “ANSi”, and “ANRi”, respectively) in replicate lines derived from the early‐flowering *B. pendula* variety BPM5. This variety starts producing inflorescences during the first growing season, and is receptive to stable transformation (Lemmetyinen et al., 1998). The RNAi constructs based on the coding sequences of *BpDFR1*, *BpANS* and *BpANR* (Methods S1) were transferred into BPM5 (hereafter the “control line”) by *Agrobacterium*‐mediated gene transfer as described in Kosonen et al. (2015) and Thitz et al. (2020). Coding sequences of *BpDFR1, BpANS* and *BpANR* correspond to genomic sequences Bpev01.c0161.g0065, Bpev01.c0717.g0023, and Bpev01.c0162.g0023, respectively, of *B. pendula* (Salojärvi et al., 2017). We used RT‐qPCR to confirm the decreased expression of the silenced genes in leaves and stems of RNAi‐modified plant lines (Table S1), using the Birch 18S ribosomal RNA gene (EMBL accession number AJ279693) as the internal reference gene. The primers used for RT‐qPCR have been published in Thitz et al. (2020) and Kosonen et al. (2015). Three to four lines per RNAi construct (for the selected lines, see label in Figure S1) with decreased condensed tannin levels in preliminary acid butanol assays (see Analytical methods) in leaves and stems of micropropagated plantlets were selected for this study.

The control and transformed lines were maintained on Murashige and Skoog culture (Murashige & Skoog, 1962) and rooted as in Thitz et al. (2020). The plants were planted on a 1:1 mixture of fertilized peat and vermiculite on April 27–28, and transferred to a greenhouse with lamps giving about 400 µmol m^‒2^ s^‒1^ additional light between 05:00–23:00 to acclimate them to greenhouse conditions. The acclimation period started close to the beginning of thermal growing season in Joensuu in eastern Finland, where the study was conducted. The plants were moved in 1.6 L pots with 2:1 mixture of peat and vermiculite 13 days before the experiment started. During this time, they were fertilized three times with a solution containing 248.6 mg/L N, 97.4 mg/L P and 561.41 mg/L K, with pots retaining circa 220 ml of solution.

### 2.2 Experimental setup

Out of 10 experimental plants from each of the four DFRi lines, four ANSi lines, three ANRi lines, and the control line, five plants were randomly allocated to either ambient or enhanced UVB treatment (altogether 120 plants). A transparent polyester panel impermeable to UVB divided the greenhouse into two compartments, one treated with ambient dose of UVB (corresponding to daily 3.82 kJ/m^2^, which is the ambient daily dose in summer in the corresponding latitude; Aphalo et al., 2012) and the other treated with a +32% enhanced dose of UVB (corresponding to daily 5.03 kJ/m^2^). Once a week, the doses received by the compartments, as well as the plants allocated into these treatments, were switched from one compartment into the other, and the location of plants in their respective compartments was randomized. The experimental plants were surrounded by extra plants, not included in the measurements.

The irradiation treatments, started on June 13, were given on 51 days around noon by UV lamps (TL40W/12 RS SLV, Philips) covered with 0.115 mm cellulose diacetate filters (Kotelo‐Rauma Ltd.) impermeable to UVC (100–280 nm). The cellulose diacetate filters were pre‐burned for 4 hr to stabilize their spectral transmittance properties, and were replaced every three weeks. The UV lamps also emit UVA, so in order to equalize the doses of UVA in the ambient and enhanced UVB treatments, a 0.175 mm UVB‐blocking polyester filter (Thermoplast) was placed over the lamps in the ambient treatment after 85 min of daily irradiation, and the lamps over both ambient and enhanced UVB compartments were turned on for another 27 min.

At the beginning of the experiment, the experimental plants had grown on soil for seven weeks, and the plants of the control line were 37.8 ± 2.5 cm (mean ± standard deviation) in height. During the experiment, the temperature in the greenhouse varied between 15.7 ± 0.7°C and 28.1 ± 3.6°C, and relative humidity between 45 ± 8% and 87 ± 5% (means and standard deviations of daily minima and maxima). A distance of 60 cm between the lamps and the top of the canopy was maintained by lifting the UV lamps and the smallest plants. The plants were watered with clean tap water, with no fertilization given during the experiment. To prevent insect proliferation, plants were sprayed with insecticide containing 0.25 g/kg cyflutrin and 0.4 g/kg transflutrin (Baygon, SC Johnson) on July 20.

### 2.3 Measurements and sampling

Stem height and basal diameter were measured and the number of dead plants were recorded weekly (altogether eight times) throughout the experiment. Development of the leaf chlorophyll content index (CCI) in mature leaves was measured with a CCM‐200 chlorophyll content meter (Opti‐Sciences) six times at 4–11 days intervals. Using the same leaves, gas exchange parameters (net photosynthetic rate A_n_ and transpiration rate E) at 25°C and 1,200 µmol m^−2^ s^−1^ PAR were measured with a LC‐PRO+ photosynthesis meter (ADC BioScientific Ltd.) between 8:30–11:00 and 13:00–15:30 twice over the experimental period (July 10–11 and July 25–26) so that on each date, the order of measurements alternated between the two treatments, and plants within the same treatment were measured in random order. For leaves in which leaf area (LA) was smaller than the aperture of the leaf chamber, estimated leaf areas were used to correct the values for A_n_ and E. Estimates for LA were means of LA in leaves from plants of the same line subjected to same treatment, sampled on August 5. Instantaneous water use efficiency (WUE) of plants was calculated as A_n_/E. Dark‐adapted photosystem II efficiency (Fv/Fm), a parameter that measures the maximum electron transferring capacity of photosynthetic light reactions, was determined with a FluorPen (Photon System Instruments, Czech Republic) after 20 min dark‐adaptation between 8:30–11:00 on two consecutive days (July 14–15 and July 27–28), with alternating order of measurements between the two treatments, and in random order within treatments.

Samples for leaf and stem phenolics and condensed tannins, as well as samples for trichome density measurements, were taken after 51 days of UVB treatments. After measuring the fresh weight (FW), samples for chemical analysis and the remaining aboveground biomass were dry‐air dried at room temperature (Tegelberg et al., 2018), while the samples for trichome density measurements were stored in +4°C and processed within 24 days. After measuring the FW and LA using a LICOR LI‐3000C portable area meter (LICOR), leaves sampled for trichome analysis were prepared into microscope samples (Thitz et al., 2017). To estimate trichome densities on upper (adaxial) and lower (abaxial) leaf surfaces, we counted glandular and hairy trichomes visible within the viewed area (0.24 cm^2^) located midway between the midrib and leaf edge with a Zeiss Stemi DV4 microscope. Resin glands visible on 2‐cm long half‐cylinders of stem were counted from photos of stems taken on August 1. For calculating total dry weight of leaf biomass, the dry weight of freshly processed samples (DW_1_) was approximated based on their fresh weight (FW_1_) and the water content of leaves sampled for chemical analyses from the same plants (WC_2_), using the formulas DW_1_ = (1 − WC_2_) · FW_1_ and WC_2_ = (FW_2_ − DW_2_)/FW_2_, where FW_2_ and DW_2_ refer to the fresh and dry weights of chemistry leaves, respectively.

Mortality during the experiment occurred only among DFRi plants, where nine individuals died in the ambient and eight individuals in the enhanced UVB treatment before the end of the experiment (Figure S3). Due to this and fragility of some of the plants, we were not able to measure all variables from all 120 plants included in the initial setup. Number of plants measured or samples taken are specified in figure legends.

### 2.4 Analytical methods

Methanol‐soluble low molecular weight (LMW) phenolics and condensed tannins were extracted from 6 to 7 mg of leaves and 7 to 8 mg of stems according to Nybakken et al. (2012). Samples were re‐dissolved in 0.6 ml of 1:1 mixture of methanol and MilliQ‐H_2_O and analyzed with a reversed‐phase HPLC‐UV‐DAD system as in Randriamanana et al. (2014). For small leaves, the method was modified to use 4–4.5 mg leaves and 0.4 ml of methanol‐water. D‐(–)‐salicin (Aldrich‐Chemie) was used as an internal standard by randomly adding it to half of the replicate samples each day. LMW phenolics were identified with UHPLC‐Q‐TOF/MS (Randriamanana et al., 2014), and quantified from data collected at 220, 280, and 320 nm on HPLC‐UV‐DAD using commercial or purified standards (Thitz et al., 2020, Methods S2, Table S2). Soluble condensed tannins from the methanol extract and insoluble condensed tannins from the extraction residue were quantified with the acid butanol assay (Hagerman, 2011a), using condensed tannins purified from the leaves of the unmodified control line as a standard (Hagerman, 2011b).

For qualitative analysis of condensed tannins from the control, ANSi and ANRi lines, approximately equal DW of leaves or stems from five plants belonging to the same line and treatment were pooled. Dry leaf samples were homogenized as in Nybakken et al. (2012) and stem samples were cut into very small pieces using scissors. Condensed tannins from 10 to 15 mg of material were extracted with a method from Scioneaux et al. (2011). Previous work suggested that some lines would contain very little condensed tannin, so we adapted the extraction method to use 30 mg of material, and increased the volumes of 2:1 chloroform:methanol (v/v) and ethyl acetate to 375 µl instead of 250 µl.

For thiolysis, 10 µl of 32% HCl in methanol and 24 µl of 5% toluene‐α‐thiol in methanol (v/v) were added into 100 µl of extract. Unreacted samples were correspondingly prepared by adding 34 µl of methanol into 100 µl of extract. Both the thiolysis and unreacted samples were incubated at +40°C for 40 min and transferred to −20°C (Scioneaux et al., 2011). To quantify the terminal and extender units of flavan‐3‐ols in condensed tannins, 10 µl of each reaction mix was separated using a gradient of 0.13% trifluoroacetic acid (TFA) in water (v/v) and 0.1% TFA in acetonitrile (v/v) (Scioneaux et al., 2011). Chromatography was carried out with a Thermo Hypersil Gold C8 column (4.5 × 150 mm, with 3 µm packing). Terminal and extender flavan‐3‐ols were identified based on their retention times and UV spectra and quantified at 220 nm (Methods S3). For each sample, free flavan‐3‐ols were determined using 10 µl of the unreacted sample, so that the amount of free flavan‐3‐ols could be subtracted from the amount of terminal flavan‐3‐ols detected in the thiolysis sample (Methods S3). The molar ratios of the terminal and extender subunits were used to calculate the average proportion of catechin‐type (trans‐%), galloylated (galloyl‐%), cyanidin‐type (CY‐type) and delphidinin‐type (DE‐type) subunits, and the mean degree of polymerization (mDP) for the condensed tannins in each sample. The average molecular weight (MW) was calculated from the mDP. This method enabled us to measure the abundance of each subunit in relation to all condensed tannin molecules present in the sample. We note that somewhat different extraction methods were used to obtain samples for the acid butanol assay and thiolysis, so slightly different fractions of the tannins may have been captured in each analysis.

### 2.5 Statistical analysis

Main and interactive effects of RNAi and UVB on chemical and morphological variables (Table S3) measured at the end of the experiment were studied with linear mixed models (e.g. Mehtätalo & Lappi, 2020) using packages lme4 (Bates et al., 2015) and lmerTest (Kuznetsova et al., 2017) in R ver. 3.5.1 (R Core Team, 2018). Random intercepts for plant lines (*a_i_*) were included in the models with the following structure:

$$y_{\mathrm{ij}}={\beta^{\prime }x}_{\mathrm{ij}}+a_{i}+\varepsilon_{\mathrm{ij}}$$

Random effects and models residuals (*ε_ij_*) were assumed to be normally distributed with a mean of zero and to have constant variance among groups. Dependent variables (*y_ij_*) were square‐root or log‐transformed (Table S3) when it clearly improved these assumptions. When significant interactive effects of RNAi and UVB were found (Table S3), we used contrasts with Holm‐adjustment for multiple comparisons (package multcomp; Hothorn et al., 2008) to test whether the RNAi‐modified plants differed from the control line in the ambient UVB treatment, and whether there were differences in responses to UVB treatments among RNAi constructs (significant differences shown in text). The interactive effect of RNAi and UVB was removed from the fixed part of the final model (***β*** *’* ***x*** *_ij_*) if it did not improve model fit in conditional *F*‐tests at *p < .05*. In case of significant main effects of RNAi and/or UVB treatment (Table S3), we used corresponding contrasts to test whether the RNAi‐modified plants differed from the control line or whether the enhanced UVB treatment differed from the ambient UVB treatment (significant differences shown in text).

Models for variables with repeated observations on same individual plants (stem height and diameter, leaf CCI, A_n_, E, and Fv/Fm) included random intercepts for plant individual (*b_ij_*) nested in the plant line (*a_i_*), which was found sufficient for modeling the dependency among the observations.

$$y_{\mathrm{ijk}}={\beta^{\prime }x}_{\mathrm{ijk}}+a_{i}+b_{\mathrm{ij}}+\varepsilon_{\mathrm{ijk}}$$

Stem height and E were square‐root transformed to satisfy the distribution and variance assumptions of random effects and model residuals described above. For initial models of stem height and diameter and leaf CCI (at least three repeated observations), initial values were included as covariates, and week (since the beginning of the experiment) and week^2^, together with their interactions with RNAi and UVB were added into the model as fixed effects (Methods S4). For initial models on gas exchange and variable fluorescence (measured twice from each plant), week, time (as hours since midnight) and time^2^ were included into the model to account for possible nonlinear responses of photosynthesis to the time of measurement. Interactive terms not improving model fit in conditional *F*‐tests (*p* > 0.05) were removed, resulting in the final model structures specified in Methods S4. *F*‐tests were used to determine which of the remaining fixed factors had statistically significant effect on modelled variable. Post‐hoc results from repeated‐effect models for A_n_, E and Fv/Fm were obtained with corresponding contrasts as described above, when significant main or interactive effects of RNAi and UVB were found.

Mortality of DFRi plants by the last growth measurement was investigated with a mixed effect logistic regression model (package lme4), using random intercepts for plant lines. *Z*‐test for the regression coefficient was used to test whether mortality differed in enhanced compared to ambient UVB treatment. No plants among the control or other RNAi‐modified lines died during the experiment.

Variability in leaf and stem phenolic composition of experimental plants was visualized with non‐parametric multidimensional scaling (NMS) in PC‐ORD ver. 7.04 (McCune & Mefford, 2016). NMS based on Sorensen distances was run with the slow‐and‐thorough autopilot option of PC‐ORD, with no penalty for tie removal. Ordinations were based on concentrations of 36 leaf and 30 stem phenolics (individual HPLC‐quantified compounds with protocatechuic acid excluded, and soluble and insoluble condensed tannins, relativized by dividing each concentration by its maximum), or abundances of 11 condensed tannin subunits (relativized as before). Blocked multi‐response permutation procedure (MRBP) based on Euclidean distances and using median alignment between blocks was applied on relativized means in each combination of RNAi and UVB treatments (8 means per compound) to test whether the phenolic composition or condensed tannin subunit composition in leaves or stems was affected by RNAi (UVB treatments used as blocks) or UVB (RNAi used as blocks). To investigate potentially different UVB responses in different RNAi constructs, we analyzed the effect on UVB on the composition of phenolics or subunits of condensed tannins in different RNAi constructs also separately. For each RNAi construct, mean concentrations of all compounds present in that construct were calculated for each combination of UVB treatment and plant line (4–8 means per construct per compound, Table S4). Then MRBP was applied on relativized means as before to test whether the composition of phenolics or subunits of condensed tannins in leaves or stems was affected by UVB (plant lines used as blocks).

Number of unique plant lines (biological replicates) per RNAi construct or UVB treatment was used in calculating standard errors (SEMs). A schematic illustration of the experimental setup, primary data and R scripts used for statistical analyses is available in an online repository (https://doi.org/10.6084/m9.figshare.13026497).

## 3 RESULTS

### 3.1 Leaf and stem phenolics

**FIGURE 2:**
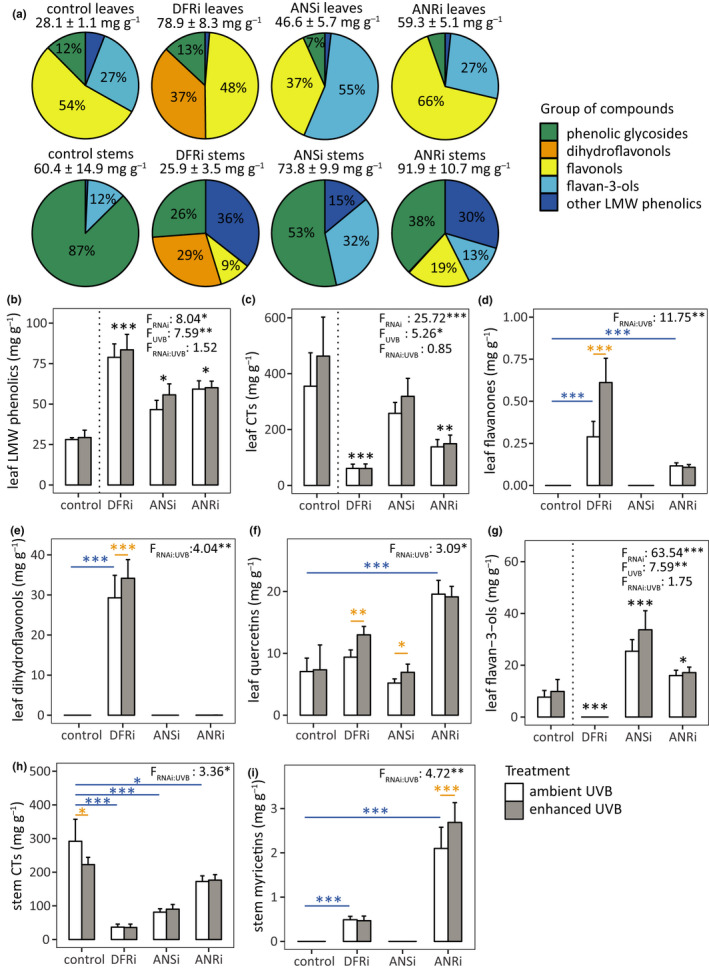
Responses of lowmolecular weight (LMW) phenolics and condensed tannins (CTs) to RNA interference and enhanced UVB. (a) Composition and total concentration (mean ± SEM) of LMW phenolics under ambient UVB treatment, from 51 control and RNAi‐modified plants. (b–i) Concentrations (mean ± SEM) of phenolic groups affected by UVB from 102 control and RNAi‐modified plants, with *F*‐values for main and interactive effects of RNAi and UVB shown. For significant interactive effects, differences from the control line in ambient UVB treatment (stars above blue lines) and differences between treatments among RNAi constructs (stars above orange lines) are shown. For significant main effects of RNAi (indicated by a dashed vertical line), RNAi constructs different from the control line are shown. *p* < 0.001 (***), 0.001 < *p* < 0.01 (**), and 0.01 < *p* < 0.05 (*). Note the differences in scales

At the end of the experiment, concentration of total LMW phenolics in leaves of the unmodified control line grown under ambient UVB treatment was about 50% of the total LMW phenolics in stems (Table 1). At the same time, leaves of the same plants had about 20% more condensed tannins compared to the stems (Table 1). LMW phenolics in the leaves of the control line were mainly flavonols (myricetins, quercetins, and kaempferols) and flavan‐3‐ols, whereas the stem LMW phenolics in the control line were dominated by phenolic glycosides (Figure 2a). The majority of condensed tannins in both leaves and stems were in the soluble fraction (Figure S2).

**TABLE 1:** Total concentrations (mg/g DW) of main phenolic groups in leaves and stems of control and RNAi‐modified plants grown under ambient (A) or enhanced UVB dose (UVB), with means (and SEM) shown. Interactive effects of RNA interference and UVB treatment (RNAi × UVB, Table S3) are described in the main text. Groups significantly different from the control line (RNAi effect) or from the ambient UVB treatment (UVB effect) are indicated, when significant main effects of RNAi or UVB were found (Table S3). For results of individual phenolics compounds, see Table S6

Compound group	control		DFRi		ANSi		ANRi		RNAi effect			UVB effect
	A	UVB	A	UVB	A	UVB	A	UVB	DFRi	ANSi	ANRi	
**LEAF LMW PHENOLICS**	**28.1 (1.1)**	**29.3 (4.6)**	**78.9 (8.3)**	**83.5 (9.5)**	**46.6 (5.7)**	**55.7 (6.8)**	**59.3 (5.1)**	**60.1 (4.1)**	↑***	↑*	↑*	↑*
Phenolic acids	1.5 (0.1)	1.4 (0.3)	0.5 (0.1)	0.5 (0.0)	0.8 (0.1)	0.7 (0.2)	0.8 (0.2)	0.7 (0.2)				
Phenolic glycosides	3.5 (0.8)	3.2 (1.0)	10.3 (1.7)	9.8 (1.9)	3.1 (0.7)	3.3 (0.7)	3.2 (0.4)	3.3 (0.5)	↑*			
Flavanones	—	—	0.3 (0.1)	0.6 (0.1)	—	—	0.1 (0.0)	0.1 (0.0)	RNAi × UVB			
Flavones	0.1 (0.1)	0.1 (0.1)	0.3 (0.0)	0.3 (0.1)	0.1 (0.0)	0.1 (0.0)	0.1 (0.0)	0.1 (0.0)				
Dihydroflavonols	—	—	29.3 (5.6)	34.2 (4.7)	—	—	0.0 (0.0)	0.0 (0.0)	RNAi × UVB			
Flavonols	15.3 (2.3)	14.8 (3.9)	38.2 (4.0)	38.1 (5.3)	17.3 (2.0)	17.9 (2.8)	39.1 (4.6)	38.8 (3.5)	↑***		↑***	
Myricetins	7.3 (1.4)	6.5 (1.1)	27.3 (4.4)	23.7 (4.8)	11.3 (1.7)	10.1 (1.8)	18.8 (2.5)	19.0 (2.5)	↑***		↑**	
Quercetins	7.1 (2.2)	7.3 (4.0)	9.4 (1.2)	13.0 (1.4)	5.2 (0.7)	6.9 (1.3)	19.6 (2.2)	19.1 (1.7)	RNAi × UVB			
Kaempferols	1.0 (0.2)	0.9 (0.2)	1.6 (0.3)	1.5 (0.2)	0.7 (0.2)	0.9 (0.2)	0.7 (0.2)	0.6 (0.2)				
Flavan‐3‐ols	7.7 (2.6)	9.9 (4.6)	—	—	25.4 (4.5)	33.7 (7.4)	16.0 (2.0)	17.1 (2.1)	↓***	↑***	↑*	↑*
**LEAF CONDENSED TANNINS**	**355.1 (119.8)**	**462.9 (139.6)**	**60.9 (15.3)**	**60.6 (16.2)**	**258.0 (38.9)**	**318.8 (64.5)**	**137.9 (26.5)**	**149.2 (30.9)**	↓***		↓**	↑*
Soluble CT	266.8 (113.8)	355.3 (140.0)	7.7 (1.2)	7.6 (1.4)	191.0 (39.4)	256.3 (64.5)	109.6 (20.7)	117.5 (23.9)	↓***		↓***	↑*
Insoluble CT	88.3 (12.8)	107.6 (15.3)	53.2 (14.2)	53.0 (14.9)	67.0 (8.1)	62.5 (8.5)	28.3 (6.4)	31.7 (8.6)			↓*	
**STEM LMW PHENOLICS** ^a^	**60.4 (14.9)**	**43.2 (7.5)**	**25.9 (3.5)**	**27.4 (35.8)**	**73.8 (9.9)**	**74.6 (11.8)**	**91.9 (10.7)**	**93.3 (11.0)**	↓*		↑*	
Phenolic acids^a^	0.4 (0.3)	0.3 (0.3)	8.6 (1.6)	10.1 (2.7)	10.2 (3.7)	10.6 (5.6)	26.6 (4.1)	25.1 (4.5)			↑***	
Phenolic glycosides	59.2 (13.3)	37.7 (7.1)	6.8 (0.9)	6.1 (1.0)	39.5 (4.8)	38.9 (5.7)	34.9 (4.8)	35.5 (5.7)	↓***			
Flavanones	—	—	0.6 (0.1)	0.6 (0.1)	—	—	0.1 (0.0)	0.1 (0.0)	↑***		↑***	
Dihydroflavonols	—	—	7.4 (1.4)	7.9 (2.2)	—	—	0.2 (0.1)	0.2 (0.0)	↑***		↑***	
Flavonols	—	—	2.5 (0.2)	2.6 (0.4)	—	—	17.3 (2.3)	19.3 (2.1)	↑***		↑***	
Myricetins	—	—	0.5 (0.1)	0.5 (0.1)	—	—	2.1 (0.5)	2.7 (0.4)	RNAi × UVB			
Quercetins	—	—	1.8 (0.2)	2.0 (0.4)	—	—	15.6 (2.1)	16.6 (1.9)	↑***		↑***	
Kaempferols	—	—	0.2 (0.0)	0.1 (0.0)	—	—	—	—	↑***			
Flavan−3‐ols	7.2 (1.8)	5.8 (0.5)	—	—	24.8 (2.8)	24.9 (3.2)	12.0 (2.1)	12.7 (1.8)	↓***	↑***	↑**	
**STEM CONDENSED TANNINS**	**292.0 (65.1)**	**222.7 (21.3)**	**36.8 (9.0)**	**35.8 (10.1)**	**81.4 (10.1)**	**90.3 (13.9)**	**172.3 (17.0)**	**176.4 (16.5)**	**RNAi × UVB**			
Soluble CT	240.2 (54.9)	186.9 (18.7)	7.0 (2.0)	6.8 (2.5)	72.2 (9.7)	80.5 (12.9)	153.4 (15.1)	155.8 (15.5)	↓***	↓***	↓***	
Insoluble CT	51.8 (10.7)	35.8 (8.1)	29.9 (7.5)	29.0 (7.8)	9.2 (0.8)	9.8 (1.4)	18.9 (3.2)	20.6 (3.6)	RNAi × UVB			

Note

SEMs were calculated as $\mathrm{SD}/\sqrt{n}$, where *SD* is the corrected sample standard deviation, and *n* (number of biological replicates) depends on the RNAi construct (*n* = 1 for the control line, *n* = 3 for the ANRi lines, and *n* = 4 for the DFRi and ANSi lines).

LMW phenolics, low molecular weight phenolics; CT, condensed tannins; –, not detected; ***, *p* < .001; **, 0.001 < *p* < .01; *, 0.01 < *p* < .05 from contrasts with Holm‐adjustment, based on linear mixed models (Table S3) done for each group of compounds.

^a^ Excluding protocatechuic acid not quantified in all plants.

RNAi had significant main effects on several groups of compounds and individual compounds (Table 1, Table S3). In leaves, decreased expression of *BpDFR1*, *BpANS,* and *BpANR* increased total LMW phenolics by 78%–183% compared to the control line (Table 1; values shown in text are averages of ambient and enhanced UVB treatments until otherwise stated). In stems, levels of total LMW phenolics decreased by 49% in DFRi plants and increased by 79% in ANRi plants compared to the control line but were not altered in ANSi lines (Table 1). Foliar condensed tannin levels decreased by 65%–85% in DFRi and ANRi lines compared to the control line (Table 1). In stems, the soluble condensed tannins decreased by 28%–97% in all RNAi plants compared to the control line (Table 1).

The effects of RNAi on LMW phenolics in leaves were similar to the earlier results obtained from the same plant lines (Thitz et al., 2020), and stem flavonoids followed a corresponding pattern: DFRi plants accumulated dihydroflavonols and flavonols but did not accumulate flavan‐3‐ols compared to the control line (Table 1). Also, decreased *BpDFR1* expression decreased phenolic glycosides in stems compared to the control line (Table 1). In ANSi plants, only flavan‐3‐ols increased compared to the control line (Table 1). Reduced *BpANR* expression increased flavonols, flavan‐3‐ols and stem phenolic acids compared to control line (Figure 2a, Table 1). These RNAi‐induced changes in phenolics caused the overall phenolic composition of both leaves (*p* = .012) and stems (*p* = .014) to change compared to the control line (Figure S1a,b).

Enhanced UVB had significant main effects on several phenolic compounds in leaves (Table S3). In leaves, total LMW phenolics increased on average by 9% (*p* = .013) and condensed tannins by 20% (*p* = .044) in all plant lines under the enhanced compared to the ambient UVB dose. The magnitude of the increase varied depending on the plant line, with increases in foliar LMW phenolics between 1%–20% and increases in foliar CT between 0%–30% (Figure 2b,c). Among different groups of leaf LMW phenolics, flavan‐3‐ols increased on average by 25% in all plant lines (*p* = .012) under the enhanced compared to the ambient UVB dose with the specific increases in different RNAi‐modified plants ranging between 0%–33% (Figure 2g). Enhanced UVB did not affect phenolic acids, flavones, myricetins or kaempferols in leaves (Table 1, Table S3). These changes under UVB enhancement caused the overall phenolic composition of leaves to change compared to the plants treated with ambient UVB dose (*p* = .039; Table S4), although the relative effect of UVB enhancement on foliar phenolics was smaller than that of RNAi (Figure S1a).

UVB had an interactive effect with RNAi on foliar flavanones (*p* < .001), dihydroflavonols (*p* = .010) and quercetins (*p* = .031; Table S3). Leaf flavanones were present exclusively in DFRi and ANRi plants and dihydroflavonols in DFRi plants (Table 1). Foliar flavanones were further increased by 112% (Figure 2d) and dihydroflavonols by 17% (Figure 2e) in DFRi plants under enhanced compared to the ambient UVB dose (*p* < .001 for both). Foliar quercetins increased by 38% in DFRi leaves (*p* = .004) and by 33% in ANSi leaves (*p* = .032) under enhanced UVB compared to their respective concentrations in the ambient UVB treatment, but there was no difference between UVB treatments in ANRi leaves (Figure 2f). When using MRBP to statistically test for the effects of UVB in each RNAi construct separately, enhanced UVB had an effect on phenolic composition of DFRi leaves (*p* = .038) but not in ANSi or ANRi leaves (Table S4). Despite minor differences between the sets of foliar phenolics used for NMS ordinations and MRBP, DFRi plants grown under enhanced UVB are slightly shifted toward lower NMS1 coordinates compared to DFRi plants grown under ambient UVB (Figure S1a).

UVB did not affect total LMW phenolics in stems (Table S3). Enhanced UVB did not have main effects on groups of phenolics or the overall phenolic composition (Figure S1b) in stems (Table S3, Table S4), but it had an interactive effect with RNAi on stem myricetins (*p* = .004), and total as well as insoluble condensed tannins (*p* = .023 and *p* = .012, respectively). Stem myricetins, present exclusively in DFRi and ANRi plants, were increased by 28% under enhanced UVB in ANRi plants compared to the corresponding plants in ambient UVB treatment (*p* < .001; Figure 2i). As expected, diminishing *BpDFR1, BpANS* or *BpANR* expression resulted in decreased stem condensed tannins (*p* < .001, *p* < .001, and *p* = .011, respectively) by 41%–87% compared to the control line under ambient UVB treatment (Figure 2h). Enhanced UVB decreased condensed tannins in stems of the control line by 24% (*p* = .015) compared to the same plants under ambient UVB treatment, but UVB treatment did not affect condensed tannins in stems of RNAi‐modified plants (Figure 2h). Insoluble CTs were decreased in ANSi stems by 82% (*p* = .007) compared to the control line under ambient UVB dose (Figure S2d). Enhanced UVB decreased insoluble CTs in stems of the control line by 31% (*p* = .012) compared to the same plants in ambient UVB treatment (Figure S2d).

### 3.2 Condensed tannin quality

Foliar condensed tannins extracted from the unmodified control line were, on average, composed of seven flavan‐3‐ol subunits, with mostly catechin as terminal subunit and a mixture of epigallocatechin, epicatechin and catechin as extenders (Table 2). Stem condensed tannins had mDP of 12–13, with catechin as terminal and epicatechin as extender subunits (Table 2). Thus, foliar condensed tannins in the control line of *B. pendula* can be classified as mixed procyanidin/prodelphinidin, whereas the stem condensed tannins were mainly procyanidins.

**TABLE 2:** Qualitative effects of RNAi on condensed tannins. Values shown are means (and SEM) from 2 to 8 samples per RNAi construct, combined from ambient and enhanced UVB treatments unless otherwise noted. Condensed tannin quality could not be detected from DFRi samples due to low yield

leaf condensed tannins	control (*n* = 2)	ANSi (*n* = 8)	ANRi (*n* = 6)
MW	2035 (51)	861 (89)***	928 (177)***
trans‐%	25.9 (0.6)	88 (2.7)***	93.6 (7.7)***
galloyl‐%	1.4 (0.3)	5.5 (1.3)	11.3 (1.5)***
CY‐type	58.6 (2.9)	67.3 (4.7)	84.5 (3)***
mDP	7.0 (0.1)	3.1 (0.4)***	3.5 (0.7)**
**stem condensed tannins**	**control (*n* = 2)**	**ANSi (*n* = 4)**	**ANRi (*n* = 4)**
MW	3,621 (30)	918 (35)***	1688 (217)***
trans‐%^a^	7.4	86.4 (0.2)***	61.3 (13.9)**
galloyl‐%	1.9 (2.7)	21.1 (1.4)	20.7 (4)
CY‐type	96.2 (0.4)	92.7 (0.4)	92.5 (1.8)
mDP	12.5 (0)	3.1 (0.1)***	5.7 (0.8)***

Note

SEMs were calculated as $\mathrm{SD}/\sqrt{n}$, where *SD* is the corrected sample standard deviation, and *n* (number of biological replicates, i.e. unique samples pooled by combining material from up to 5 replicate plants) depends on the RNAi construct and plant part as shown in column headings, with the exception of trans‐% in stem condensed tannins.^a^

MW, mean molecular weight (mg/mol); trans‐%, average proportion of catechin‐type subunits (%); galloyl‐%, average proportion of subunits esterified by gallic acid (%); CY‐type, average proportion of cyanidin‐type subunits (%); mDP, mean degree of polymerization; ***, *p* < .001; **, 0.001 < *p* < .01; *, 0.01 < *p* < .05 from contrasts with Holm‐adjustment, based on linear mixed models in Table S3.

^a^ In the ambient UVB treatment; *n* = 1 for the control line, *n* = 2 for ANSi and ANRi lines.

Decreased *BpANS* and *BpANR* expression had profound effects on the structural features of condensed tannins in both leaves and stems (Table 2, Table S3). Structural features of condensed tannins in DFRi plants could not be defined due to low yield of condensed tannins from DFRi samples. Reduced ANS or ANR activity reduced mDP by about 50% in leaves and stems per an average condensed tannin molecule, and increased trans‐% from 7%–26% to 61%–94% (*p* < .01 for all effects; Table 2). In ANRi leaves, the proportion of galloylated and cyanidin‐type subunits also increased compared to the control line (*p* < .001 for both, Table 2). The overall subunit composition of condensed tannins was affected by RNAi in leaves (*p* = .047, Figure S1c), but in stems, the effect of RNAi was only marginally significant (*p* = .062; Figure S1d).

Enhanced UVB increased the proportion of cyanidin‐type subunits in leaf condensed tannins by 11% in all plants (*p* = .018; Figure 4a). No other main effects of UVB on the condensed tannin structure (Table S3) or subunit composition (Table S4) were found. UVB and RNAi had a statistically significant interactive effect on trans‐% in stem CTs (Table S3): ANSi and ANRi stems had more catechin‐type subunits than the stems of the control line when grown under the ambient UVB dose (*p* < .001 and *p* = .006, respectively), whereas the control stems grown under enhanced UVB had higher trans‐% compared to the same plants in the ambient UVB treatment (*p* < .001). However, it should be noted that the samples from each line and treatment were pooled before the CT quality analysis, meaning that we had a single sample per UVB treatment from the control line. When using MRBP to test for the effects of UVB on tannin quality in ANRi and ANSi leaves and stems separately, enhanced UVB had a significant effect on subunit composition of condensed tannins in ANSi leaves (*p* = .037) but not in ANSi stems or ANRi leaves or stems (Table S4).

### 3.3 Growth and photosynthesis

**FIGURE 3:**
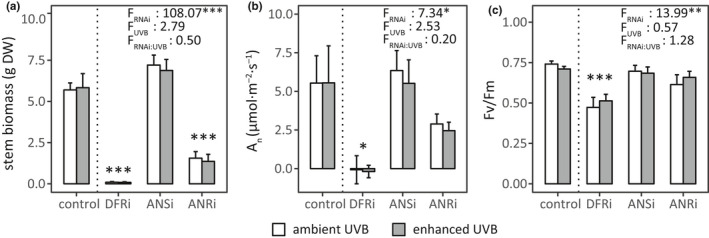
Effects of RNAi on (a) final stem biomass, (b) net photosynthetic rate A_n_, and (c) maximum quantum yield of photosystem II (Fv/Fm). Means and SEMs of (a) 102 plants, (b) 2 measurements from 79–99 plants, or (c) 2 measurements from 85–102 plants grown under ambient and enhanced UVB are shown. *F*‐values related to main and interactive effects of RNAi and UVB are indicated, as well as RNAi constructs different from the control line at *p* < .001 (***), 0.001 < *p* < .01 (**), and 0.01 < *p* < .05 (*)

RNAi had significant main effects on final leaf and stem biomass (dry weight of stems in Figure 3a; leaves followed a similar pattern). Development of stem height and basal diameter strongly depended on initial differences between RNAi‐modified plants, and their curvilinear development during the experiment (modeled with a quadratic function; Figure S3a,b) depended on RNAi (Table S5). Development of stem basal diameter was additionally affected by UVB treatment (Figure S3b, Table S5). By the final biomass measurements, DFRi plants had 98% lower stem and 94% lower leaf biomass than the control line (*p* < .001 for both), whereas ANRi decreased biomass of stems by 75% (*p* < .001, Figure 3a) and that of leaves by 51% (*p* < .001; values shown are averages of both UVB treatments). The final leaf or stem biomasses of ANSi plants did not differ from the control line (Figure 3a; leaf biomass not shown). UVB did not affect final leaf or stem biomasses (Table S3) or stem height (Table S5). There were no interactive effects of RNAi and UVB on any of the measured growth parameters (Table S3, Table S5).

Similar to growth results, photosynthetic variables A_n_, Fv/Fm, and WUE were affected by RNAi, while UVB treatment had no effects (Table S3). These parameters were suppressed in DFRi plants compared to the control line (*p* = .016, *p* < .001, and *p* < .001, respectively), whereas in ANSi and ANRi plants they did not differ from the control line (*p* = .220–0.999, Figure 3b,c, Figure S4b). Foliar CCI (Figure S3c) strongly depended on initial values, and the curvilinear development of foliar CCI during the experiment depended on RNAi (modelled with a quadratic function; Table S5). Enhanced UVB caused a modest but uniform decrease in leaf CCI compared to the ambient UVB treatment, but the response was not affected by RNAi or week of measurement (Table S5). Unlike the other photosynthetic variables, UVB treatment and RNAi had significant interactive effects on transpiration rate (E; Table S3). Under the ambient UVB treatment, DFRi decreased E compared to the control line (*p* < .001), but the corresponding value in ANRi and ANSi plants did not differ from the control line (Figure S4a). Enhanced UVB reduced E in ANSi plants (*p* < .001), but did not affect E in other RNAi‐modified plants (Figure S4a).

By the end of the experiment, there was considerable mortality (42.5%) among DFRi plants, but no plants died in the control line or other RNAi‐modified plants. In DFRi plants, there was no difference in mortality between different UVB treatments (*Z* = −0.39, *p* = .700; lower panel of Figure S3a).

### 3.4 Leaf trichomes and stem resin glands

**FIGURE 4:**
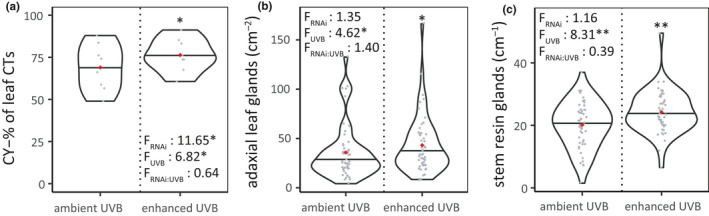
(a) Proportion of cyanidin‐type subunits in leaf CTs, or density of (b) glandular trichomes on the upper surface of 101 upper leaf surfaces and (c) resin glands visible on 1 cm of 99 stems from plants grown under ambient or enhanced UVB treatments. Density distribution, median (line) and mean (red dot), as well as original data (gray dots) are shown. *F*‐values related to main and interactive effects of RNAi and UVB are indicated, as well as significant differences from the ambient UVB treatment at *p* < .001 (***), 0.001 < *p* < .01 (**), and 0.01 < *p* < .05 (*)

The density of glandular or hairy trichomes or resin glands was not affected by RNAi (Table S3). At the end of the experiment, plants treated with enhanced UVB had a higher density of glandular trichomes on the upper leaf surface (*p* = .032, Figure 4b) and higher density of resin glands in their stems (*p* = .004; Figure 4c) compared to plants in the ambient UVB treatment. Hairy leaf trichomes or glandular trichomes on the lower leaf surface were not affected by UVB (Table S3).

## 4 DISCUSSION

### 4.1 Flexible leaf phenolics and morphological responses indicate resilience to enhanced UVB

Concern for very high UVB exposure has been greatly relieved since international treaties started controlling the use of ozone‐depleting substances (WMO, 2018), but continuing emissions from existing sources may slow down the positive development in ozone recovery (Lickley et al., 2020). Additionally, seasonal variability in the total ozone layer is likely to continue causing episodic high‐irradiance events in high latitudes (Bednarz et al., 2016; Manney et al., 2011). During those events, UVB‐levels may exceed the tolerance limits of organisms. The enhanced UVB doses of this study correspond to about 20% ozone decrease (e.g. Nybakken et al., 2012) projected in central Finland, if the use of ozone‐depleting substances had not been banned.

In this study, many of the measured growth and photosynthetic parameters did not decrease under +32% UVB enhancement (Tables S3, S5). This is consistent with meta‐analyses evaluating plant responses to moderate UVB supplements (Li et al., 2010; Searles et al., 2001), and indicates that the morphological and/or chemical defenses in the focal early‐flowering variety of *B. pendula* were sufficient to overcome the harmful effects of UVB. UVB‐specific accumulation of phenolics, frequently reported for both woody and herbaceous species (Demkura et al., 2010; Randriamanana et al., 2015) was clearly seen in leaf samples collected after more than 7 weeks of UVB exposure (Figure 2b).

Partial inhibition of the flavonoid pathway by RNA interference determined the exact compounds that accumulated under enhanced UVB. For example in DFRi leaves, in which the production of both types of condensed tannin subunits was impeded (Figure 1d; for more detailed pathway, see James et al., 2017), enhanced UVB led to accumulation of several flavonoid intermediates that were missing from the leaves of the unmodified control plants (Table 1). Thus, UVB‐related induction of flavanones and dihydroflavonols in DFRi leaves was probably caused by blockage of the condensed tannin pathway (Figure 2c), which led to the accumulation of alternative end products (Figure 2d,e). UVB‐related induction of quercetins, a group of flavonol glycosides with dihydroxylated B‐rings, is consistent with results from earlier studies on *B. pendula* (Lavola et al., 2013) and other woody and herbaceous species (Hofmann et al., 2003; Nissinen et al., 2017). Quercetins accumulated in control, DFRi and ANSi leaves, whereas in ANRi leaves, which have constitutively high levels of flavonol glycosides (Figure 2a; Kosonen et al., 2015; Thitz et al., 2020), enhanced UVB treatment did not further increase the levels of leaf quercetins (Figure 2e).

Phenolic acids did not accumulate under UVB‐enhancement in *B. pendula*, which corresponds to the results of Keski‐Saari et al. (2005) but contrasts with other reports (Lavola et al., 2000). These compounds are generally very variable over time, and between genotypes, species and light environments (Bidel et al., 2007; Lavola et al., 2000).

Enhanced UVB induced minor chemical changes in stems (Figure 2i, Table S3). Sivadasan et al. (2018) obtained similar results in a UVB experiment with woody tissues and bark from *Populus tremula* L. Weak or non‐existent UVB induction of phenolics in samples mainly composed of woody tissues suggests that the stem tissues sensitive to UVB are primarily protected by bark, not by phenolics.

Potential morphological defenses that increased under enhanced UVB levels in this study include phenolic‐containing stem resin glands and glandular trichomes on upper leaf surface (Figure 4b,c; Taipale et al., 1994; Valkama et al., 2004). Earlier studies conducted with *B. pendula* suggest that UV radiation affects gland density at least in this species (Kostina et al., 2001; Robson et al., 2015). Reports on other plant species show that high light or supplemental UVB can affect the density or biosynthesis of leaf glands (Barnes et al., 1996; Tattini et al., 2000). The increased density of glandular trichomes under enhanced UVB supports the hypothesis that they may have UVB‐protective functions in addition to their antiherbivore properties in *B. pendula* (Martemyanov et al., 2015; Thitz et al., 2020).

### 4.2 Could condensed tannins have photoprotective roles in *B. pendula*?

Our study showed that early‐flowering *B. pendula* accumulates foliar condensed tannins under +32% UVB enhancement (Figure 2c). Reported effects of UVB on condensed tannins in leaves of *B. pendula* range from increased levels (Kotilainen et al., 2009) to no significant effects (Tegelberg et al., 2001). UVB‐related increases in condensed tannins have been found in other woody species including *Picea abies* Karst. (Virjamo et al., 2014) and *Populus trichocarpa* Torr. & Gray (Mellway & Constabel, 2009), but not in *P. tremula* (Randriamanana et al., 2015). Clearly, UVB‐induced accumulation of condensed tannins is more variable across taxa than is the well‐established increase in flavonols in leaves of woody plants.

UVB‐related accumulation of flavonoid compounds, especially those with di‐ or trihydroxylated B‐rings (Figure 1a), may be a consequence of their potential to scavenge reactive oxygen species (Agati & Tattini, 2010). Our finding of an increase in the relative abundance of dihydroxylated CY‐type subunits in polymeric condensed tannins under enhanced UVB provides to our knowledge the first evidence of structural changes in condensed tannins as a result of environmental stress (Scioneaux et al., 2011). This structural change in foliar condensed tannins is not expected to increase their efficiency in photoprotection, since dihydroxylated CY‐type and trihydroxylated DEL‐type structures have equal antioxidative capacities (Rice‐Evans et al., 1996). However, increased levels of foliar condensed tannins could still improve their overall role as antioxidants under enhanced UVB.

Taken together, our results do not provide conclusive evidence for the photoprotective role of condensed tannins. Growth and photosynthesis in the control and RNAi‐modified plants responded similarly to UVB enhancement (Tables S3 or Table S5), indicating that the plants that survived until the end of the experiment were equally resistant to enhanced UVB, regardless of changes in polyphenols resulting from pathway blockages. The condensed tannin accumulation we observed under enhanced UVB could be an indirect effect of the UVR8‐mediated upregulation of the whole flavonoid‐tannin pathway (Kliebenstein et al., 2002), but the increased condensed tannins do not appear to confer resistance to UVB enhancement. However, considering the earlier suggestions that hyperaccumulation of condensed tannins improves resilience to UVB in MYB134‐overexpressing poplars (Mellway & Constabel, 2009), our finding about the general vulnerability of the very‐low tannin DFRi plants indicates that the physiological consequences of altered condensed tannin metabolism deserve further study.

### 4.3 Maintaining the balance between flavonoid and condensed tannin production may be important for normal growth in early‐flowering *Betula pendula*

Unlike *Populus* sp., *B. pendula* has only one functional copy of *ANR* in its genome (Kosonen et al., 2015). Accumulation of *trans*‐flavan‐3‐ols in ANSi leaves and stems (Table 1) supports the interpretation that partial silencing of *BpANS* caused an accumulation of its substrates, which the leucoanthocyanidin reductase (LAR) converted into catechin and gallocatechin (Figure 1d). The decreased growth of flavonoid‐accumulating DFRi and ANRi plants (Figures 2a and 3a) is consistent with earlier experiments using the same plant material (Kosonen et al., 2015; Thitz et al., 2020). Similar chemical changes (increased flavonoids or decreased condensed tannins) have been linked with decreased growth in *A. thaliana* with silenced lignin biosynthesis (Besseau et al., 2007) and in a DFR‐deficient mutant of *Nicotiana tabacum* L. (Kazama et al., 2013). Besides their multiple roles in plant defense, flavonoid aglycones have the capacity to inhibit polar auxin transport (Besseau et al., 2007; Brown et al., 2001; Peer et al., 2004), which may at least partially explain the stunted growth of the DFRi and ANRi plants compared to the control line.

Considering that DFRi and ANRi leaves have similar levels of foliar flavonoids, the more severe growth inhibition in the tannin‐deficient DFRi (Figure 3a) suggests that condensed tannins might be essential for normal growth. This is supported by the mortality of the DFRi plants under both ambient and realistically elevated UVB doses (Figure S3a). For example, decreased photosystem II efficiency and net photosynthesis in the DFRi plants (Figure 3b,c) indirectly supports the proposal that condensed tannins participate in regulation of the oxidative state of photosynthetic cells (Gourlay & Constabel, 2019; Harding, 2019). This functionality would fit the suggested polymerization of condensed tannins in chloroplast‐derived tannosomes (Brillouet et al., 2013) and the recently identified links between polyphenol oxidation and acute oxidative stress in chloroplasts of *Helianthus annuus* L. (Samson et al., 2020). Alternatively, decreased growth and increased mortality of DFRi plants could be explained by a structural role for insoluble condensed tannins that are bound to the polysaccharide matrix of cell walls (Renard et al., 2001). The normal growth phenotype of ANSi plants (Figure 3a) and MYB‐overexpressing poplars that accumulate both flavonoids and condensed tannins (James et al., 2017; Mellway et al., 2009), lead us to propose that normal plant growth requires a balance between flavonoid and condensed tannin production.

To conclude, we found an increase in foliar condensed tannins of *B. pendula* under enhanced UVB similarly to some other studies. However, our results from genetically modified plants suggest that condensed tannins have at most a minor photoprotective role in *B. pendula*, because decreased levels of condensed tannins in DFRi plants did not lead to decreased performance under UVB enhancement. Additionally, our study shows that structural features of condensed tannins can change in response to environmental stress. Beyond the photoprotective and antiherbivore roles of flavonoids and condensed tannins (e.g. Julkunen‐Tiitto et al., 2005; Thitz et al., 2020), our results suggest that balanced production of these compounds is paramount for normal growth. Implications of this finding for the architecture and distribution of these protective compounds in plants requires further study.

### CONFLICT OF INTEREST

The authors declare no conflict of interest.

[Correction added on 11 June 2021, after first online publication: Conflict of Interest statement added to provide full transparency.]

## Supporting information

Supplementary Material
