# Supplementary material for: Genetic modification of the flavonoid pathway alters growth and reveals flexible responses to enhanced UVB – Role of foliar condensed tannins
Source: Plant Environ Interact. 2020 Dec 28;2(1):1–15. doi: 10.1002/pei3.10036 (PMC10168092; doi:10.1002/pei3.10036)
Supplement: Supplementary file 1 — Supplementary Material [file PEI3-2-1-s001.pdf]

Supplementary material to ”Modification of the flavonoid pathway alters growth and reveals flexible responses to enhanced UVB - role of foliar condensed tannins”

Authors: Thitz, P., Hagerman, A.E., Randriamanana, T.R., Virjamo, V., Kosonen, M., Lännenpää, M., Nyman, T., Mehtätalo, L., Kontunen-Soppela, S., Julkunen-Tiitto, R.

Journal: Plant-Environment Interactions

This supplementary file includes following items.

Figure S1. NMS ordinations of phenolics and condensed tannin structure in leaves and stems

Figure S2. Soluble and insoluble fractions of condensed tannin in leaves and stems

Figure S3. Growth and leaf chlorophyll development during experiment

Figure S4. Evaporation and water use efficiency

Table S1. Expression of *BpDFR1*, *BpANS* or *BpANR* after the transformation

Table S2. Identification and quantification of stem LMW phenolics

Table S3. F-tests related to interactive and main effects of RNAi and UVB on response variables

Table S4. MRBP testing the effect of RNAi and UVB on phenolic composition

Table S5. F-tests related to interactive and main effects of RNAi and UVB on growth and leaf chlorophyll content

Table S6. Concentrations of individual phenolics

Methods S1. The coding sequences of putative *BpDFR1*, *BpANS*, and *BpANR* genes

Methods S2. Identification of leaf LMW phenolics not described in Thitz et al. (2020)

Methods S3. Flavan-3-ol subunits of condensed tannins identified from thiolysis samples

Methods S4. Initial and final fixed effect structures of linear mixed effect models with repeated measurements

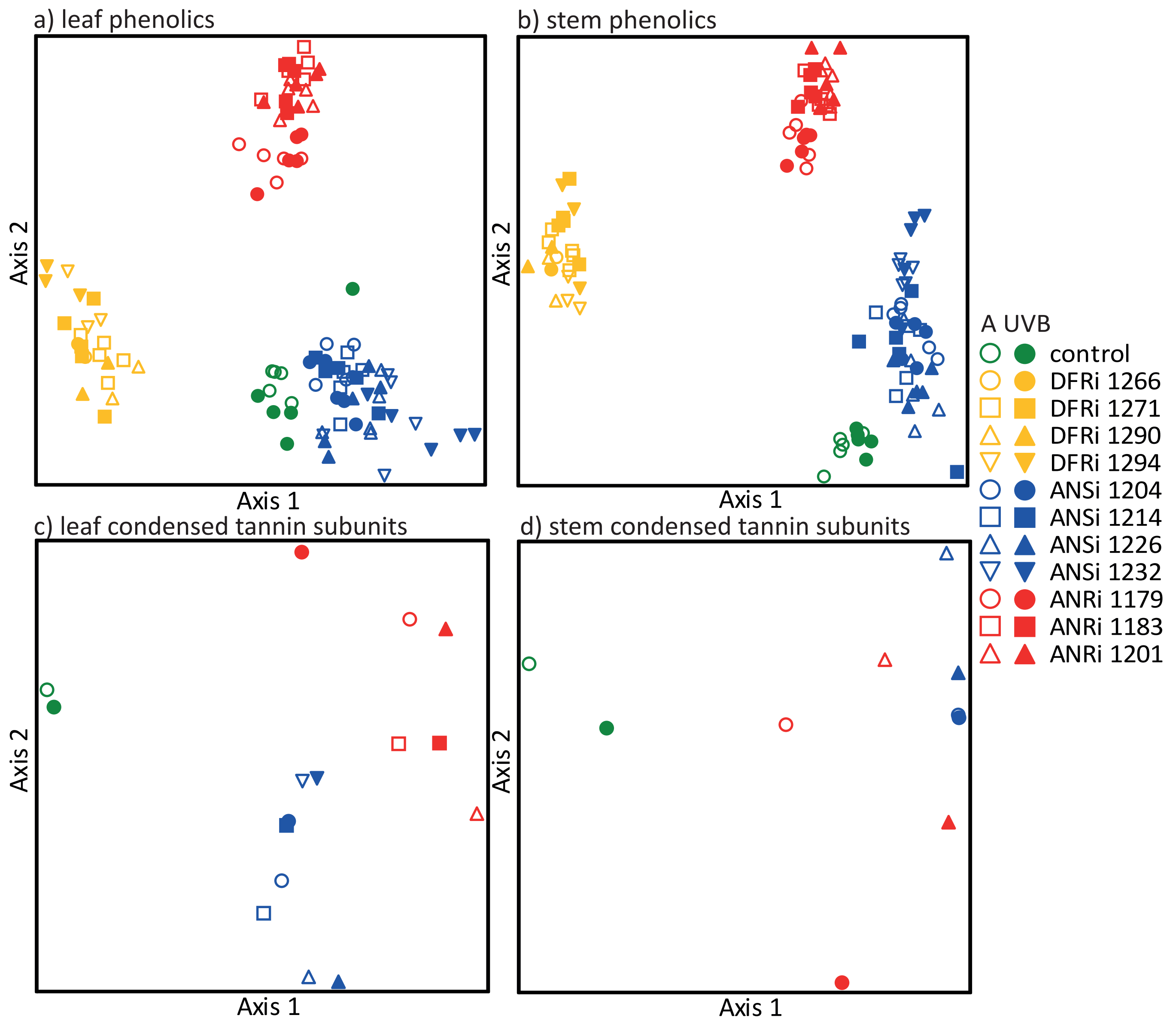

Figure S1. NMS ordinations for concentrations of a) 36 leaf compounds (stress=9.901) and b) 30 stem compounds (stress=6.876) measured from 102 plants, and c) for relative abundances of 10 condensed tannin subunits in 16 leaf samples (stress=8.792) and d) 9 condensed tannin subunits in 10 stem samples (stress=2.203). For c and d, each sample was pooled from 5 replicate plants belonging to the same plant line and UVB treatment (A, ambient UVB dose; UVB, enhanced UVB dose). Subunit content of condensed tannins could not be defined from DFRi samples due to low yield.

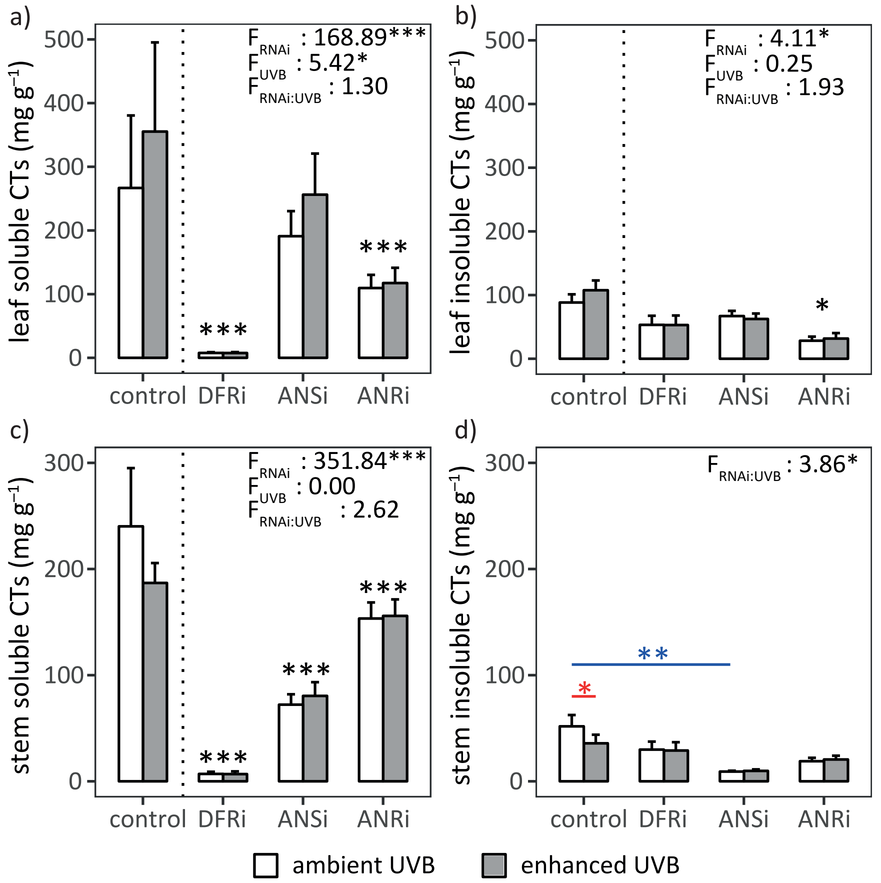

Figure S2. Soluble (a, c) and insoluble (b, d) condensed tannin (CT) concentration in leaves (a, b) and stems (c, d). Means and SEMs from 102 control and RNAi-modified plants grown under ambient or enhanced UVB treatments are shown, with F-values for main and interactive effects of RNAi and UVB shown. For significant interactive effects, differences from the control line in ambient UVB treatment (stars above blue lines) and differences between treatments among RNAi constructs (stars above red lines) are shown. For significant main effects of RNAi (indicated by dashed vertical line), RNAi constructs different from the control line are shown. P<0.001 (***), 0.001<P<0.01 (**), and 0.01<P<0.05 (*).

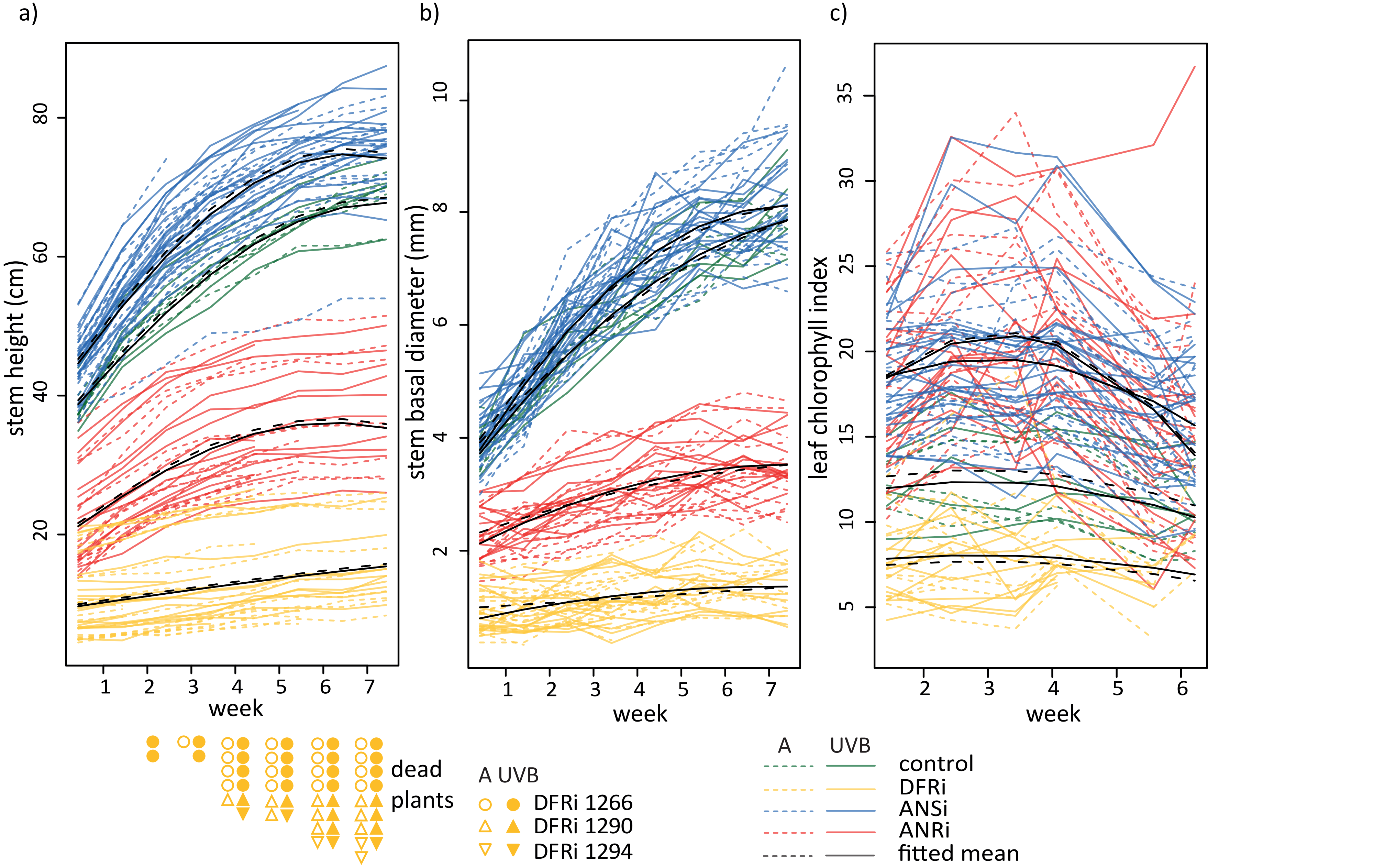

Figure S3. Development of a) stem height and b) basal diameter, and c) leaf chlorophyll index during over 7 weeks of ambient (A) or enhanced UVB (UVB) treatments. Original data measured from 120 (a, b) or from 113 control and RNAi-modified plants plants (c), and fitted means for each combination of RNAi construct and UVB treatment from mixed effect models (Table S5) are shown. Cumulative number of dead DFRi plants by each week is shown below x-axis of Fig. S3a.

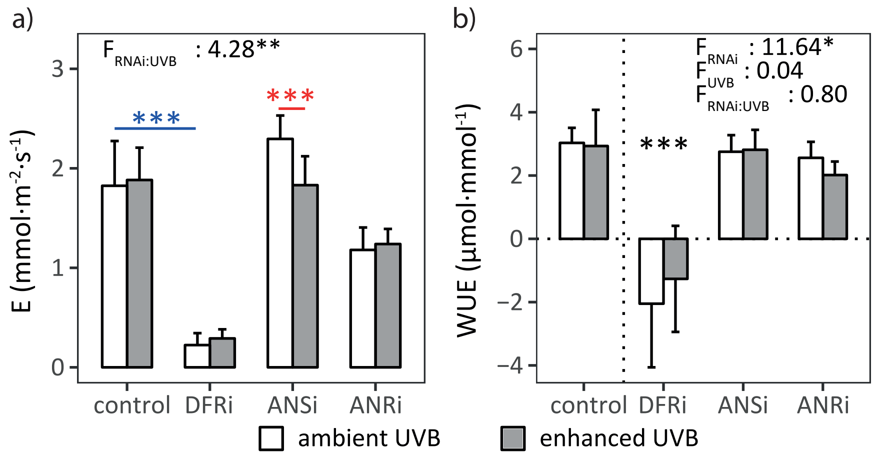

Figure S4. a) Transpiration (E) and b) instantaneous water use efficiency (An / E) in different RNAi-modified plants and UVB treatments. Means and SEMs based on 2 measurements from 79–99 plants, with F-values for main and interactive effects of RNAi and UVB shown. For significant interactive effects, differences from the control line in ambient UVB treatment (stars above blue lines) and differences between treatments among RNAi constructs (stars above red lines) are shown. For significant main effects of RNAi (indicated by dashed vertical line), RNAi constructs different from the control line are shown. P<0.001 (***), 0.001<P<0.01 (**), and 0.01<P<0.05 (*).

Table S1. Remaining expression of silenced genes after the transformation, measured with RT-qPCR from 6–8 independent transgenic lines per RNAi construct.

RNAi construct	remaining expression compared to the control line	
	in leaves†	in stems
DFRi	9.6 – 31.7 %	1.1 – 23.6 %
ANSi	1.2 – 18.9 %	1.8 – 10.3 %
ANRi	5.7 – 47.8 %	3.1 – 84.8 %

RNAi, RNA interference; DFR, dihydroflavonol reductase; ANS, anthocyanidin synthase; ANR, anthocyanidin reductase. Remaining expression in each RNAi line was calculated as expression of the silenced gene relative to the expression of the internal reference gene (means of three technical replicates per line). Maxima and minima of remaining expression in RNAi lines are shown relative to the mean expression of the same gene in the control sample (means of three technical replicates).

†Earlier published in Thitz et al. (2020).

Table S2**.** Identification of 29 low-molecular phenolics with UHPLC-Q-TOF/MS from the stems of the control and modified *Betula pendula* lines used in the experiment, and wavelengths and standards used for quantification with HPLC-UV-DAD. Compounds were identified based on mass from UHPCL-QTOF/MS and, when this was unavailable, on UV-spectra from HPLC-UV-DAD.

		HPLC-UV-DAD			UHPLC-QTOF/MS			
	Compound	Rt, min	λ, nm	Standard	Rt, min	Monoisotopic mass	Measured mass	ppm
S1	protocatechuic acid monoglucoside	1.1	220	protocatechuic acid	3.363	339.0692‡	339.0681	-3.26
S2	protocatechuic acid†	2.2	220	protocatechuic acid				
S3	gentisic acid derivative	3.2	320	protocatechuic acid				
S4	rhododendrin derivative 1	3.5	280	platyphylloside				
S4F	dihydroflavonol 1	3.7	280	ampelopsin				
S4A	rhododendrin derivative 2	4.1	280	platyphylloside				
S4C	a lignan/neolignan	4.4	220	(+)-catechin				
S4E	3,4'-dihydroxypropiophenone-3-glucoside (DPPG)	4.9	280	picein				
S4D	coumaroylquinic acid derivative 3	8.7	320	*p*-OH-cinnamic acid	3.513	339.1080§	339.1049	-9.13
S4B	flavanone 1	9.1	280	eriodictyol				
S5/6	procyanidin B3	9.2	220	(+)-catechin	4.412	579.1503§	579.1492	-1.82
S7	catechin	10.2	220	(+)-catechin	4.878	291.0869§	291.0872	1.15
S8	ampelopsin monoglucoside	11.6	280	ampelopsin	5.311	505.0958‡	505.0961	0.56
S10	rhododendrin	12.4	280	platyphylloside	6.777	351.1420‡	351.1411	-2.49
S9	ampelopsin	13.0	280	ampelopsin	5.500	321.0610§	321.0623	3.91
S10B	dihydroflavonol 2	14.6	280	ampelopsin				
S10C	taxifolin monoglucoside	15.7	280	taxifolin	7.293	489.1009‡	489.1009	0.00
S10D	taxifolin	17.6	280	taxifolin	7.326	305.0661§	305.0656	-1.64
S11	myricetin 3-galactoside	18.6	320	myricetin 3-rhamnoside	8.080	503.0802‡	503.0795	-1.32
S12	myricetin 3-glucoside	18.9	320	myricetin 3-rhamnoside	8.192	503.0802‡	503.0796	-1.12
S14	quercetin 3-galactoside	21.3	320	quercetin 3-galactoside	9.158	487.0853‡	487.0863	2.16
S15	quercetin 3-glucoside	21.8	320	quercetin 3-galactoside	9.308	487.0853‡	487.0852	-0.10
S16+17	quercetin 3-arabinoside	22.6	320	quercetin 3-galactoside	9.674	457.0747‡	457.0738	-1.94
S18	platyphylloside	24.0	280	platyphylloside	10.157	499.1944‡	499.1937	-1.41

	Compound	HPLC-UV-DAD Rt, min	λ, nm	Standard	UHPLC-QTOF/MS Rt, min	Monoisotopic mass	Measured mass	ppm
S19	quercetin 3-rhamnoside	24.7	320	quercetin 3-galactoside	10.340	471.0903‡	471.0897	-1.35
S20a	isorhamnetin derivative 1	25.0	320	isorhamnetin 3-glucoside	10.802	501.1009‡	501.1005	-0.80
S20c	kaempferol 3-rhamnoside	28.0	320	kaempferol 3-rhamnoside	11.623	455.0954‡	455.0949	-1.14
S21	platyphylloside derivative 1	34.5	280	platyphylloside				
S22	platyphylloside derivative 2	35.0	280	platyphylloside				

Rt, retention time; λ, wavelength of quantification; ppm, mass accuracy (10^6 x (monoisotopic mass – measured mass)/(measured mass).

†Protocatechuic acid overlapped with salicin added as an internal standard in HPLC-UV-DAD chromatograms, and thus could not be reliably quantified from these samples. Thus, statistical analysis of protocatechuic acid is limited to samples without added salicin (n=48 observations).

‡$(M+Na)^+$

§$(M+H)^+$

Table S3. F-tests for interactive and main effects of RNAi and UVB on response variables. Linear mixed effect models included random intercepts for plant line (the control line; four DFRi lines; four ANSi lines; three ANRi lines). Interactive term RNAi x UVB was removed when it was not significant at P<0.05 in conditional F-tests, before completing F-tests of the main effects.

	RNAi x UVB				RNAi				UVB			
	df1	df2	F	P	df1	df2	F	P	df1	df2	F	P
**Chemical variables**												
sqrt(Total leaf LMW phenolics)	3	86	1.52	0.215	3	7	8.04	0.010	1	89	7.59	0.007
log(Leaf phenolic acids+1)	3	87	0.08	0.972	3	8	3.27	0.078	1	90	1.41	0.239
p-OH-cinnamic acid monogluc.†	3	86	0.27	0.849	3	8	15.19	0.001	1	89	4.00	0.049
log(coumaroylquinic acid der. 1 + 0.1)	3	87	0.04	0.990	3	8	18.28	0.001	1	90	0.17	0.682
coumaroylquinic acid der. 2	3	85	0.45	0.717	3	6	19.34	0.001	1	88	2.53	0.115
Leaf DPPG	3	86	0.43	0.731	3	8	8.41	0.008	1	89	0.00	0.981
log(Leaf flavanones + 0.01)	3	80	11.75	0.000								
sqrt(flavanone 1)	3	82	6.11	0.001								
flavanone 2	3	94	15.75	0.000								
flavanone 3	3	79	10.98	0.000								
sqrt(Leaf flavones)†	3	86	0.92	0.437	3	8	6.54	0.015	1	89	0.03	0.860
log(flavone 1 + 0.1)	3	87	1.10	0.352	3	8	11.20	0.003	1	90	0.00	0.997
sqrt(flavone 2)†	3	86	0.76	0.519	3	8	4.77	0.035	1	89	0.03	0.853
Leaf dihydroflavonols	3	85	4.04	0.010								
dihydroflavonol 1	3	86	5.25	0.002								
ampelopsin digluc.	3	84	4.03	0.010								
ampelopsin monogluc.	3	86	8.29	0.000								
ampelopsin	3	85	2.37	0.076	3	7	20.28	0.001	1	88	1.87	0.175
dihydroflavonol 2	3	85	3.47	0.020								
taxifolin monogluc.	3	85	6.25	0.001								
log(taxifolin + 0.1)	3	85	1.07	0.368	3	6	59.96	0.000	1	88	1.68	0.199
sqrt(dihydroflavonol 3)	3	85	1.96	0.126	3	6	80.01	0.000	1	88	1.56	0.214
sqrt(Leaf flavonols)	3	86	0.10	0.958	3	8	15.77	0.001	1	89	0.00	0.947

	RNAi x UVB				RNAi				UVB			
	df1	df2	F	P	df1	df2	F	P	df1	df2	F	P
log(Leaf myricetins)	3	86	0.81	0.493	3	8	9.15	0.006	1	89	4.26	0.042
log(myr-3-gal + 1)	3	86	1.64	0.186	3	8	3.02	0.093	1	89	1.19	0.278
log(myr-3-gluc + 1)	3	86	1.67	0.180	3	8	20.37	0.000	1	89	0.44	0.509
myr-3-arab	3	86	1.20	0.316	3	8	2.08	0.180	1	89	0.48	0.490
log(myr-3-rhamn)	3	86	0.87	0.459	3	7	12.51	0.003	1	89	4.02	0.048
log(methylmyr-3-gluc+1)	3	86	1.26	0.294	3	8	48.99	0.000	1	89	2.71	0.103
sqrt(Leaf quercetins)	3	87	3.09	0.031								
quer-3-gal	3	86	0.65	0.587	3	8	0.12	0.948	1	89	7.01	0.010
sqrt(quer-3-gluc)	3	86	0.59	0.624	3	8	74.21	0.000	1	89	0.01	0.905
log(quer-3-arab + 0.1)	3	85	1.23	0.305	3	7	149.05	0.000	1	88	1.62	0.206
log(quer-3-rhamn)†	3	87	2.84	0.043								
quercetin der. 1	3	88	0.03	0.992	3	8	376.88	0.000	1	90	0.04	0.834
Foliar kaempferols†	3	86	2.09	0.107	3	8	4.55	0.039	1	89	0.08	0.774
kaemp-3-rhamn	3	86	2.53	0.063	3	8	1.81	0.225	1	89	1.37	0.244
sqrt(dicoum. astragalin)	3	86	0.90	0.445	3	8	5.10	0.030	1	89	0.00	0.995
kaempferol der. 1	3	86	1.74	0.165	3	8	2.44	0.140	1	89	0.79	0.376
log(Leaf flavan-3-ols + 10)	3	87	1.75	0.162	3	8	63.54	0.000	1	90	7.59	0.007
sqrt(gallocat. der. 1)	3	87	0.05	0.986	3	8	177.42	0.000	1	90	0.11	0.742
log(gallocat. der. 2 + 1)	3	87	0.25	0.862	3	8	112.34	0.000	1	90	0.77	0.382
log(catechin + 1)	3	87	1.32	0.274	3	8	148.39	0.000	1	90	6.54	0.012
log(proanthocyanidin olig. + 0.1)	3	87	1.50	0.221	3	8	93.17	0.000	1	90	5.28	0.024
log(Leaf condensed tannins)	3	86	0.85	0.470	3	8	25.72	0.000	1	89	5.26	0.024
log(leaf soluble CT)	3	87	1.30	0.279	3	8	168.89	0.000	1	90	5.42	0.022
log(leaf insoluble CT)	3	86	1.93	0.130	3	8	4.11	0.049	1	89	0.25	0.617
log(Total stem LMW phenolics)‡	3	86	1.59	0.198	3	7	20.57	0.001	1	89	0.32	0.571
sqrt(Stem phenolic acids)‡	3	86	0.31	0.821	3	8	5.82	0.021	1	89	0.07	0.785

	RNAi x UVB				RNAi				UVB			
	df1	df2	F	P	df1	df2	F	P	df1	df2	F	P
protoc. acid monogluc.†	3	86	2.27	0.086	3	8	4.72	0.034	1	89	0.81	0.370
sqrt(protoc. acid)	3	34	1.60	0.207	3	7	13.73	0.003	1	37	1.52	0.226
gentisic acid der. 1	3	86	0.42	0.739	3	8	5.46	0.025	1	89	0.00	0.965
coumaroylquinic acid der. 3	3	84	1.91	0.134	3	6	2.62	0.150	1	87	3.95	0.050
log(Stem phenolic glycosides + 10)	3	87	1.45	0.234	3	7	89.61	0.000	1	89	1.36	0.247
sqrt(rhodod. der. 1)†	3	86	3.80	0.013								
sqrt(rhodod. der. 2)†	3	86	0.83	0.482	3	8	6.06	0.018	1	89	0.71	0.401
sqrt(DPPG)	3	86	0.76	0.521	3	8	32.66	0.000	1	89	0.01	0.918
log(rhodod.)	3	87	1.17	0.324	3	8	139.09	0.000	1	90	0.76	0.386
log(platyp.)	3	85	0.66	0.578	3	7	140.58	0.000	1	88	1.34	0.251
platyp. der. 1	3	87	0.03	0.992	3	8	12.54	0.002	1	90	0.08	0.775
platyp. der. 2	3	87	0.02	0.995	3	8	6.69	0.013	1	90	0.00	0.953
log(Stem flavanone 1 + 0.1)	3	85	0.14	0.933	3	7	136.58	0.000	1	88	0.01	0.926
log(Stem dihydroflavonols + 0.1)	3	85	0.62	0.601	3	7	229.35	0.000	1	88	1.03	0.312
dihydroflavonol 1	3	85	5.82	0.001								
ampelopsin monogluc.	3	86	0.68	0.565	3	8	24.95	0.000	1	89	0.57	0.452
log(ampelopsin + 1)	3	86	0.01	0.998	3	8	38.06	0.000	1	89	0.01	0.910
dihydroflavonol 2	3	86	5.20	0.002								
taxifolin monogluc.	3	86	0.75	0.524	3	8	40.75	0.000	1	89	0.63	0.431
sqrt(taxifolin)	3	85	1.26	0.294	3	6	125.09	0.000	1	88	3.44	0.067
log(Stem flavonols + 0.1)	3	85	0.92	0.436	3	7	426.77	0.000	1	88	1.03	0.314
sqrt(Stem myricetins)	3	86	4.72	0.004								
log(myr-3-gal + 0.1)	3	84	4.81	0.004								
log(myr-3-gluc + 0.01)	3	86	2.42	0.072	3	8	181.28	0.000	1	89	2.15	0.146
log(Stem quercetins + 1)	3	85	0.56	0.644	3	7	239.66	0.000	1	88	1.48	0.228
log(quer-3-gal + 0.1)	3	85	0.79	0.504	3	7	139.69	0.000	1	88	0.64	0.425
sqrt(quer-3-gluc)	3	86	0.82	0.486	3	7	155.21	0.000	1	89	0.58	0.448

	RNAi x UVB				RNAi				UVB			
	df1	df2	F	P	df1	df2	F	P	df1	df2	F	P
log(quer-3-arab + 0.01)	3	85	1.59	0.199	3	7	71.08	0.000	1	88	0.01	0.907
sqrt(quer-3-rhamn)	3	86	1.26	0.293	3	7	67.90	0.000	1	89	0.61	0.435
log(isorhamnetin der. 1 + 0.01)	3	86	1.41	0.246	3	8	386.64	0.000	1	89	2.33	0.131
Stem kaemp-3-rhamn	3	85	2.22	0.091	3	7	27.97	0.000	1	88	1.76	0.188
sqrt(Stem flavan-3-ols)	3	87	0.52	0.671	3	8	205.36	0.000	1	90	0.23	0.632
log(procyanidin B3 + 1)	3	88	0.25	0.860	3	8	181.94	0.000	1	91	0.00	0.967
sqrt(catechin)	3	87	0.58	0.626	3	8	185.10	0.000	1	90	0.20	0.653
sqrt(stem lignan/neolignan 1)	3	86	1.01	0.394	3	8	8.95	0.006	1	89	0.58	0.450
sqrt(Stem condensed tannins)	3	85	3.36	0.023								
sqrt(stem soluble CT)	3	84	2.62	0.056	3	5	351.84	0.000	1	87	0.00	0.957
log(stem insoluble CT)	3	86	3.86	0.012								
leaf CT molecular weight	2	5	0.79	0.502	2	5	10.68	0.016	1	7	0.94	0.364
sqrt(100 - leaf CT cat-%)	2	5	2.92	0.145	2	12	15.58	0.000	1	12	0.60	0.455
leaf CT galloyl-%	2	5	0.03	0.972	2	5	10.42	0.016	1	7	0.16	0.703
leaf CT CY-type	2	5	0.64	0.564	2	5	11.65	0.013	1	7	6.82	0.035
leaf CT chain length	2	5	0.34	0.729	2	5	7.17	0.034	1	7	0.58	0.471
stem CT molecular weight	2	2	0.16	0.863	2	2	23.37	0.041	1	4	0.61	0.479
stem CT cat-%	2	2	40.92	0.024								
stem CT galloyl-%	2	2	1.30	0.435	2	2	3.95	0.202	1	4	1.07	0.359
stem CT CY-type	2	2	8.23	0.108	2	2	1.65	0.378	1	4	3.55	0.133
stem CT chain length	2	2	0.35	0.743	2	2	22.70	0.042	1	4	0.32	0.602
**Morphological variables**												
sqrt(leaf biomass)	3	86	0.81	0.494	3	8	98.75	0.000	1	89	0.77	0.383
sqrt(stem biomass)	3	86	0.50	0.682	3	8	108.07	0.000	1	89	2.79	0.098
sqrt(adaxial gland density)	3	84	1.40	0.249	3	7	1.35	0.335	1	87	4.62	0.034
abaxial gland density	3	85	0.03	0.994	3	8	2.38	0.148	1	88	1.71	0.195
adaxial hair density	3	84	0.18	0.913	3	7	0.68	0.590	1	87	1.16	0.284

	RNAi x UVB				RNAi				UVB			
	df1	df2	F	P	df1	df2	F	P	df1	df2	F	P
sqrt(abaxial hair density)	3	63	0.88	0.456	3	9	2.32	0.147	1	66	2.13	0.149
resin gland density	3	83	0.39	0.763	3	6	1.16	0.394	1	86	8.31	0.005
**Physiological variables**[§]												
Fv/Fm	3	86	1.28	0.286	3	8	13.99	0.002	1	84	0.57	0.453
A	3	95	0.20	0.896	3	8	7.34	0.011	1	89	2.53	0.115
sqrt(E)	3	90	4.28	0.007								
WUE	3	49	0.80	0.499	3	4	11.64	0.016	1	43	0.04	0.851

LMW phenolics, low-molecular weight phenolics; monogluc., monoglucoside; der., derivative; DPPG, dihydroxypropiophenone-3-glucoside; digluc., diglucoside; myr-3-gal, myricetin 3-galactoside; myr-3-gluc, myricetin 3-glucoside; myr-3-arab, myricetin 3-arabinoside; methylmyr-3-gluc; myricetin methyl ether 3-glucoside; quer-3-gal, quercetin 3-galactoside; quer-3-gluc, quercetin 3-glucoside; quer-3-arab, quercetin 3-arabinoside; quer-3-rham, quercetin 3-rhamnoside; kaemp-3-rhamn, kaempferol 3-rhamnoside; dicoum., dicoumaroyl; gallocat., gallocatechin; olig., oligomer; CT, condensed tannins; cat-%, mean proportion of catechin-type subunits in CT; protoc., protocatechuic; rhodod., rhododendrin; platyp., platyphylloside; galloyl-%, mean proportion of galloylated subunits in CT; CY-type, mean proportion of cyanidin-type subunits in CT; Fv/Fm, maximum quantum yield of photosystem II; E, evaporation rate; A; net photosynthetic rate; WUE, instantaneous water use efficiency

[†]no significant differences in Holm-adjusted contrasts

[‡]excluding protocatechuic acid

[§] Models for physiological variables included random intercept for plant individual (nested within plant line). To consider the possible non-linear relationship with time (h since midnight; modelled with quadratic function) or week of measurement, additional fixed effect terms were included. Model for Fv/Fm included an additional main effect terms for time (df1=1, df2=162, F=0.04, P=0.840), time^2 (df1=1, df2=163, F=0.04, P=0.850) and repeat (df1=1, df2=91, F=35.46, P<0.001). Model for A included additional main effect terms for time (df1=1, df2=164, F=4.64, P=0.033), time^2 (df1=1, df2=165, F=4.58, P=0.034) and repeat (df1=1, df2=91, F=0.69, P=0.41). Model for square-root transformed E included additional main effect terms for time (df1=1, df2=152, F=5.41, P=0.021), time^2 (df1=1, df2=152, F=4.44, P=0.037), and repeat (df1=1, df2=84, F=3.75, P=0.056). Model for WUE included additional main effect terms for time (df1=1, df2=133, F=2.30, P=0.131), time^2 (df1=1, df2=135, F=2.66, P=0.105), and repeat (df1=1, df2=43, F=4.96, P=0.031).

Table S4. P-values related to the effects of RNAi or UVB on composition of low-molecular weight (LMW) phenolics or condensed tannin (CT) subunits in leaves and stems, according to MRBP. Data consists of means within each combination of RNAi and UVB treatments (8 means per compound), or each combination of plant lines and UVB treatments (4–8 means per compound, depending on RNAi construct), relativized by dividing each compound/subunit by its maximum mean in that dataset.

	leaves		stems	
grouping factor	36 LMW phenolics	10 CT subunits	30 LMW phenolic†	9 CT subunits
RNAi construct	0.012	0.047	0.014	0.062
UVB treatment	0.039	0.140	0.187	0.072
within DFRi‡	0.038		0.057	
within ANSi§	0.262	0.037	0.728	0.159
within ANRi¶	0.737	0.104	0.472	0.159

†excluding protocatechuic acid

‡relativized datasets included 30 leaf compounds and 26 stem compounds measured from 4 DFRi lines

§relativized datasets included 22 leaf compounds and 15 stem compounds measured from 4 ANSi lines, 8 leaf CT subunits measured from 4 ANSi lines, and 9 stem CT subunits measured from 2 ANSi lines

¶relativized datasets included 26 leaf compounds and 24 stem compounds measured from 3 ANRi lines, 7 leaf CT subunits measured from 3 ANRi lines and 8 stem CT subunits measured from 2 ANRi lines

Table S5. F-test results from repeated effect models for stem height (L, cm), stem basal diameter (D, mm) and leaf chlorophyll content (CCI). Models included random intercepts for plant lines and individual plants nested within plant lines. Interactive terms were removed from final models when they were not significant at P<0.05 in conditional F-tests.

	sqrt(L)				D				CCI			
	df1	df2	F	P	df1	df2	F	P	df1	df2	F	P
RNAi	3	10	5.5	0.016	3	14	2.1	0.150	3	29	0.9	0.459
UVB	1	108	1.7	0.197	1	482	10.7	0.001	1	98	5.7	0.019
initial value	1	112	240.0	<0.001	1	111	89.9	<0.001	1	99	193.9	<0.001
week	1	708	548.4	<0.001	1	737	190.6	<0.001	1	475	1.0	0.314
$week^2$	1	708	163.0	<0.001	1	738	28.9	<0.001	1	475	1.9	0.165
RNAi x week	3	708	246.7	<0.001	3	740	219.2	<0.001	3	478	8.1	<0.001
RNAi x $week^2$	3	708	123.6	<0.001	3	741	59.2	<0.001	3	479	10.8	<0.001
UVB x week	1	710	0.7	0.419	1	742	11.9	<0.001	1	480	0.7	0.390
UVB x $week^2$	1	709	1.0	0.316	1	742	7.4	0.007	1	479	0.2	0.667
RNAi x UVB	3	103	0.2	0.927	3	102	0.3	0.823	3	97	0.6	0.618
RNAi x UVB x week	3	703	0.8	0.469	3	734	1.6	0.193	3	474	0.4	0.785
RNAi x UVB x $week^2$	3	702	0.4	0.768	3	735	1.2	0.322	3	475	0.3	0.790

Table S6. Concentrations (mg g^{-1} DW) of groups of phenolics (in bold) and 36 individual compounds in leaves and 29 individual compounds in stems of control and RNAi-modified plants grown under ambient (A) or enhanced (UVB) dose of UVB, with means (SEM, with n = number of lines per construct) shown. Interactive effects for RNAi and UVB (RNAi x UVB) on different groups of phenolics or individual phenolics are indicated. For groups or individual compounds with significant main effects, concentrations different from the control line (RNAi effect) or from the ambient UVB treatment (UVB effect) are indicated with direction and significance of effects. All P-values are obtained from contrasts with Holm-adjustment, based on linear mixed models described in Table S3.

	control		DFRi		ANSi		ANRi		RNAi effect			UVB effect
**group** / compound	A	UVB	A	UVB	A	UVB	A	UVB	DFRi	ANSi	ANRi	
**LEAF LMW PHENOLICS**	**28.1 (1.1)**	**29.3 (4.6)**	**78.9 (8.3)**	**83.5 (9.5)**	**46.6 (5.7)**	**55.7 (6.8)**	**59.3 (5.1)**	**60.1 (4.1)**	↑ ***	↑ *	↑ *	↑ *
**Leaf phenolic acids**	**1.5 (0.1)**	**1.4 (0.3)**	**0.5 (0.1)**	**0.5 (0.0)**	**0.8 (0.1)**	**0.7 (0.2)**	**0.8 (0.2)**	**0.7 (0.2)**				
p-OH-cinnamic acid monoglucoside	0.4 (0.1)	0.4 (0.1)	0.2 (0.0)	0.1 (0.0)	0.0 (0.0)	0.0 (0.0)	0.3 (0.1)	0.3 (0.1)	↓ ***	↓ ***		
coumaroylquinic acid der. 1†	0.7 (0.1)	0.8 (0.2)	–	–	0.7 (0.1)	0.7 (0.2)	0.5 (0.1)	0.4 (0.1)	↓ ***			
coumaroylquinic acid der. 2	0.4 (0.0)	0.3 (0.2)	0.3 (0.1)	0.3 (0.0)	0.1 (0.1)	0.0 (0.0)	0.1 (0.1)	0.0 (0.1)		↓ ***	↓ ***	
**Leaf phenolic glycosides**												
DPPG	3.5 (0.8)	3.2 (1.0)	10.3 (1.7)	9.8 (1.9)	3.1 (0.7)	3.3 (0.7)	3.2 (0.4)	3.3 (0.5)	↑ *			
**Leaf flavanones**	–	–	**0.3 (0.1)**	**0.6 (0.1)**	–	–	**0.1 (0.0)**	**0.1 (0.0)**		RNAi x UVB§		
flavanone 1	–	–	0.1 (0.0)	0.2 (0.1)	–	–	0.1 (0.0)	0.1 (0.0)		RNAi x UVB§		
flavanone 2	–	–	0.1 (0.0)	0.2 (0.0)	–	–	–	–		RNAi x UVB§		
flavanone 3	–	–	0.1 (0.0)	0.2 (0.0)	–	–	–	–		RNAi x UVB§		
**Leaf flavones**	**0.1 (0.1)**	**0.1 (0.1)**	**0.3 (0.0)**	**0.3 (0.1)**	**0.1 (0.0)**	**0.1 (0.0)**	**0.1 (0.0)**	**0.1 (0.0)**				
flavone 1	0.0 (0.0)	0.0 (0.0)	0.1 (0.0)	0.1 (0.0)	0.0 (0.0)	0.0 (0.0)	0.0 (0.0)	0.0 (0.0)	↑ *			
flavone 2	0.1 (0.0)	0.1 (0.0)	0.2 (0.0)	0.2 (0.1)	0.1 (0.0)	0.1 (0.0)	0.0 (0.0)	0.0 (0.0)				
**Leaf dihydroflavonols**	–	–	**29.3 (5.6)**	**34.2 (4.7)**	–	–	**0.0 (0.0)**	**0.0 (0.0)**		RNAi x UVB§		
dihydroflavonol 1	–	–	0.6 (0.1)	0.8 (0.2)	–	–	–	–		RNAi x UVB§		

**group** / compound	control A	control UVB	DFRi A	DFRi UVB	ANSi A	ANSi UVB	ANRi A	ANRi UVB	RNAi effect DFRi	RNAi effect ANSi	RNAi effect ANRi	UVB effect
ampelopsin diglucoside	–	–	0.1 (0.1)	0.3 (0.1)	–	–	–	–		RNAi x UVB§		
ampelopsin monoglucoside	–	–	1.8 (0.3)	2.3 (0.4)	–	–	–	–		RNAi x UVB§		
ampelopsin	–	–	24.3 (4.8)	27.3 (3.8)	–	–	–	–	↑ ***			
dihydroflavonol 2	–	–	0.6 (0.2)	0.9 (0.3)	–	–	–	–		RNAi x UVB§		
taxifolin monoglucoside	–	–	1.5 (0.2)	1.9 (0.3)	–	–	–	–		RNAi x UVB§		
taxifolin	–	–	0.2 (0.1)	0.2 (0.0)	–	–	0.0 (0.0)	0.0 (0.0)	↑ ***			
dihydroflavonol 3	–	–	0.3 (0.1)	0.4 (0.2)	–	–	–	–	↑ ***			
**Leaf flavonols**	**15.3 (2.3)**	**14.8 (3.9)**	**38.2 (4.0)**	**38.1 (5.3)**	**17.3 (2.0)**	**17.9 (2.8)**	**39.1 (4.6)**	**38.8 (3.5)**	↑ ***		↑ ***	
**Leaf myricetins**	**7.3 (1.4)**	**6.5 (1.1)**	**27.3 (4.4)**	**23.7 (4.8)**	**11.3 (1.7)**	**10.1 (1.8)**	**18.8 (2.5)**	**19.0 (2.5)**	↑ ***		↑ **	
myr-3-gal	0.6 (0.2)	0.4 (0.1)	2.3 (0.5)	3.0 (1.0)	1.3 (0.6)	1.0 (0.5)	1.3 (0.4)	1.2 (0.2)				
myr-3-gluc	0.6 (0.2)	0.4 (0.1)	2.6 (0.6)	3.0 (0.8)	1.1 (0.4)	0.9 (0.3)	8.6 (1.0)	9.1 (1.2)	↑ *		↑ ***	
myr-3-arab	–	–	0.1 (0.0)	0.1 (0.0)	0.0 (0.0)	0.0 (0.0)	0.1 (0.1)	0.2 (0.1)				
myr-3-rham	6.0 (1.2)	5.3 (1.2)	21.9 (3.8)	17.1 (3.2)	8.9 (1.2)	8.3 (1.2)	7.4 (1.2)	7.2 (1.2)	↑ ***			
methylmyr-3-gluc	0.1 (0.2)	0.1 (0.2)	0.3 (0.1)	0.4 (0.1)	–	–	1.4 (0.3)	1.4 (0.2)			↑ ***	
**Leaf quercetins**	**7.1 (2.2)**	**7.3 (4.0)**	**9.4 (1.2)**	**13.0 (1.4)**	**5.2 (0.7)**	**6.9 (1.3)**	**19.6 (2.2)**	**19.1 (1.7)**		RNAi x UVB§		
quer-3-gal	1.2 (0.6)	1.2 (1.0)	1.0 (0.3)	1.9 (0.5)	0.9 (0.3)	1.6 (0.4)	1.3 (1.0)	1.6 (0.8)				↑ **
quer-3-gluc	0.4 (0.2)	0.4 (0.3)	0.4 (0.1)	0.6 (0.2)	0.4 (0.1)	0.4 (0.2)	5.5 (1.0)	5.2 (0.6)			↑ ***	
quer-3-arab	–	0.1 (0.1)	0.1 (0.0)	0.1 (0.0)	–	–	0.9 (0.2)	0.9 (0.2)	↑ *		↑ ***	
quer-3-rham	5.5 (1.6)	5.7 (3.2)	7.9 (1.0)	10.4 (1.1)	3.9 (0.5)	5.0 (0.9)	5.5 (1.0)	5.0 (0.7)				
quercetin der. 1	–	–	–	–	–	–	6.3 (0.7)	6.4 (0.6)			↑ ***	

group / compound	control A	control UVB	DFRi A	DFRi UVB	ANSi A	ANSi UVB	ANRi A	ANRi UVB	RNAi effect DFRi	RNAi effect ANSi	RNAi effect ANRi	UVB effect
**Leaf kaempferols**	**1.0 (0.2)**	**0.9 (0.2)**	**1.6 (0.3)**	**1.5 (0.2)**	**0.7 (0.2)**	**0.9 (0.2)**	**0.7 (0.2)**	**0.6 (0.2)**				
kaemp-3-rham	0.8 (0.2)	0.8 (0.3)	0.7 (0.1)	0.8 (0.1)	0.5 (0.1)	0.7 (0.2)	0.5 (0.2)	0.4 (0.1)				
dicoum. astragalin	0.1 (0.1)	0.1 (0.1)	0.7 (0.2)	0.5 (0.1)	0.1 (0.1)	0.2 (0.1)	0.1 (0.1)	0.1 (0.1)	↑ *			
kaempferol der. 1	0.0 (0.0)	0.0 (0.0)	0.2 (0.0)	0.1 (0.0)	0.0 (0.0)	0.0 (0.0)	0.0 (0.0)	0.0 (0.0)				
**Leaf flavan-3-ols**	**7.7 (2.6)**	**9.9 (4.6)**	–	–	**25.4 (4.5)**	**33.7 (7.4)**	**16.0 (2.0)**	**17.1 (2.1)**	↓ ***	↑ ***	↑ *	↑ *
gallocatechin der. 1	0.0 (0.0)	0.0 (0.0)	–	–	1.3 (0.3)	1.4 (0.3)	–	–		↑ ***		
gallocatechin der. 2	0.4 (0.2)	0.5 (0.2)	–	–	3.8 (0.7)	4.1 (0.7)	0.1 (0.1)	0.2 (0.1)	↓ *	↑ ***		
catechin	6.6 (2.3)	8.6 (4.0)	–	–	17.0 (3.3)	22.8 (5.5)	13.6 (1.7)	14.5 (1.7)	↓ ***	↑ ***	↑ *	↑ *
proanthocyanidin oligomer	0.6 (0.2)	0.8 (0.4)	–	–	3.3 (1.0)	5.4 (2.3)	2.3 (0.4)	2.5 (0.5)	↓ ***	↑ ***	↑ **	↑ *
**LEAF CONDENSED TANNINS**	**355.1 (119.8)**	**462.9 (139.6)**	**60.9 (15.3)**	**60.6 (16.2)**	**258.0 (38.9)**	**318.8 (64.5)**	**137.9 (26.5)**	**149.2 (30.9)**	↓ ***		↓ **	↑ *
leaf soluble CT	266.8 (113.8)	355.3 (140.0)	7.7 (1.2)	7.6 (1.4)	191.0 (39.4)	256.3 (64.5)	109.6 (20.7)	117.5 (23.9)	↓ ***		↓ ***	↑ *
leaf insoluble CT	88.3 (12.8)	107.6 (15.3)	53.2 (14.2)	53.0 (14.9)	67.0 (8.1)	62.5 (8.5)	28.3 (6.4)	31.7 (8.6)			↓ *	
**STEM LMW PHENOLICS**[‡]	**60.4 (14.9)**	**43.2 (7.5)**	**25.9 (3.5)**	**27.4 (35.8)**	**73.8 (9.9)**	**74.6 (11.8)**	**91.9 (10.7)**	**93.3 (11.0)**	↓ *		↑ *	
**Stem phenolic acids**[‡]	**0.4 (0.3)**	**0.3 (0.3)**	**8.6 (1.6)**	**10.1 (2.7)**	**10.2 (3.7)**	**10.6 (5.6)**	**26.6 (4.1)**	**25.1 (4.5)**			↑ ***	
protoc. acid monoglucoside	–	–	–	–	–	0.0 (0.0)	0.0 (0.0)	0.0 (0.0)				
protoc. acid[‡]	0.1 (0.0)	0.0 (0.0)	0.1 (0.0)	0.1 (0.0)	0.1 (0.0)	0.1 (0.0)	0.3 (0.1)	0.3 (0.1)			↑ ***	
gentisic acid der. 1	0.3 (0.3)	0.3 (0.2)	8.5 (1.6)	10.1 (2.7)	10.2 (3.7)	10.6 (5.6)	26.6 (4.1)	25.0 (4.5)			↑ **	
coumaroylquinic	0.1 (0.0)	0.0 (0.0)	0.0 (0.0)	0.0 (0.0)	0.0 (0.0)	0.0 (0.0)	0.0 (0.0)	0.0 (0.0)				↓ *

**group** / compound	control		DFRi		ANSi		ANRi		RNAi effect			UVB effect
	A	UVB	A	UVB	A	UVB	A	UVB	DFRi	ANSi	ANRi	
acid der. 3												
**Stem phenolic glycosides**	**59.2 (13.3)**	**37.7 (7.1)**	**6.8 (0.9)**	**6.1 (1.0)**	**39.5 (4.8)**	**38.9 (5.7)**	**34.9 (4.8)**	**35.5 (5.7)**	↓ ***			
rhodod. der. 1	1.2 (0.2)	0.7 (0.1)	3.3 (0.7)	2.6 (0.8)	1.0 (0.2)	0.9 (0.2)	0.2 (0.2)	0.4 (0.3)				
rhodod. der. 2	0.2 (0.3)	0.2 (0.2)	0.1 (0.1)	0.0 (0.1)	1.3 (0.5)	1.5 (0.6)	2.1 (0.5)	2.3 (0.6)				
DPPG	–	–	0.8 (0.2)	1.0 (0.2)	0.0 (0.0)	0.0 (0.0)	0.1 (0.0)	0.1 (0.0)	↑ ***			
rhodod.	19.9 (6.2)	13.8 (2.5)	1.5 (0.1)	1.4 (0.2)	13.0 (1.5)	13.0 (2.2)	6.8 (1.1)	7.2 (1.2)	↓ ***		↓ ***	
platyp.	31.6 (7.5)	22.4 (5.1)	1.0 (0.2)	1.0 (0.2)	22.2 (3.2)	21.6 (3.3)	22.1 (3.8)	21.8 (4.5)	↓ ***			
platyp. der. 1	–	–	–	–	0.3 (0.2)	0.4 (0.2)	1.1 (0.2)	1.1 (0.3)			↑ ***	
platyp. der. 2	–	–	–	0.0 (0.0)	1.7 (0.6)	1.6 (0.7)	2.5 (0.4)	2.5 (0.5)			↑ *	
**Stem flavanones**												
flavanone 1	–	–	0.6 (0.1)	0.6 (0.1)	–	–	0.1 (0.0)	0.1 (0.0)	↑ ***		↑ ***	
**Stem dihydroflavonols**	–	–	**7.4 (1.4)**	**7.9 (2.2)**	–	–	**0.2 (0.1)**	**0.2 (0.0)**	↑ ***		↑ ***	
dihydroflavonol 1	–	–	0.2 (0.0)	0.2 (0.0)	–	–	–	–		RNAi x UVB§		
ampelopsin monoglucoside	–	–	0.7 (0.1)	0.7 (0.1)	–	–	–	–	↑ ***			
ampelopsin	–	–	5.2 (1.2)	5.6 (1.9)	–	–	–	–	↑ ***			
dihydroflavonol 2	–	–	0.3 (0.0)	0.4 (0.1)	–	–	–	–		RNAi x UVB§		
taxifolin monoglucoside	–	–	0.5 (0.1)	0.5 (0.1)	–	–	–	–	↑ ***			
taxifolin	–	–	0.5 (0.1)	0.6 (0.1)	–	–	0.2 (0.1)	0.2 (0.0)	↑ ***		↑ ***	
**Stem flavonols**	–	–	**2.5 (0.2)**	**2.6 (0.4)**	–	–	**17.3 (2.3)**	**19.3 (2.1)**	↑ ***		↑ ***	
**Stem myricetins**	–	–	**0.5 (0.1)**	**0.5 (0.1)**	–	–	**2.1 (0.5)**	**2.7 (0.4)**		RNAi x UVB§		
myr-3-gal	–	–	0.2 (0.0)	0.2 (0.1)	–	–	1.2 (0.3)	1.7 (0.3)		RNAi x UVB§		
myr-3-gluc	–	–	0.3 (0.0)	0.3 (0.1)	–	–	0.9 (0.2)	1.0 (0.2)	↑ ***		↑ ***	

group / compound	control A	control UVB	DFRi A	DFRi UVB	ANSi A	ANSi UVB	ANRi A	ANRi UVB	RNAi effect DFRi	RNAi effect ANSi	RNAi effect ANRi	UVB effect
**Stem quercetins**	–	–	**1.8 (0.2)**	**2.0 (0.4)**	–	–	**15.6 (2.1)**	**16.6 (1.9)**	↑ ***		↑ ***	
quer-3-gal	–	–	0.5 (0.1)	0.5 (0.1)	–	–	3.4 (0.8)	3.9 (0.8)	↑ ***		↑ ***	
quer-3-gluc	–	–	0.5 (0.1)	0.5 (0.1)	–	–	3.7 (0.5)	4.0 (0.6)	↑ ***		↑ ***	
quer-3-arab	–	–	0.1 (0.0)	0.1 (0.0)	–	–	0.6 (0.1)	0.6 (0.1)	↑ ***		↑ ***	
quer-3-rhamn	–	–	0.3 (0.1)	0.4 (0.1)	–	–	0.8 (0.1)	0.8 (0.2)	↑ ***		↑ ***	
isorhamnetin der. 1	–	–	0.4 (0.1)	0.5 (0.1)	–	–	7.1 (1.2)	7.3 (1.0)	↑ ***		↑ ***	
**Stem kaempferols**	–	–	**0.2 (0.0)**	**0.1 (0.0)**	–	–	–	–				
kaemp-3-rhamn	–	–	0.2 (0.0)	0.1 (0.0)	–	–	–	–	↑ ***			
**Stem flavan-3-ols**	**7.2 (1.8)**	**5.8 (0.5)**	–	–	**24.8 (2.8)**	**24.9 (3.2)**	**12.0 (2.1)**	**12.7 (1.8)**	↓ ***	↑ ***	↑ **	
procyanidin B3	0.9 (0.1)	1.0 (0.1)	–	–	2.1 (0.2)	2.0 (0.2)	0.6 (0.1)	0.6 (0.1)	↓ ***	↑ ***	↓ **	
catechin	6.3 (1.9)	4.8 (0.5)	–	–	21.9 (2.8)	22.8 (3.3)	11.4 (2.0)	12.2 (1.8)	↓ ***	↑ ***	↑ ***	
Other compounds												
lignan/neolignan 1	0.0 (0.0)	0.0 (0.0)	0.1 (0.0)	0.1 (0.0)	0.1 (0.0)	0.1 (0.1)	0.4 (0.1)	0.4 (0.1)			↑ ***	
**STEM CONDENSED TANNINS**	**292.0 (65.1)**	**222.7 (21.3)**	**36.8 (9.0)**	**35.8 (10.1)**	**81.4 (10.1)**	**90.3 (13.9)**	**172.3 (17.0)**	**176.4 (16.5)**		RNAi x UVB§		
soluble CT	240.2 (54.9)	186.9 (18.7)	7.0 (2.0)	6.8 (2.5)	72.2 (9.7)	80.5 (12.9)	153.4 (15.1)	155.8 (15.5)	↓ ***	↓ ***	↓ ***	
insoluble CT	51.8 (10.7)	35.8 (8.1)	29.9 (7.5)	29.0 (7.8)	9.2 (0.8)	9.8 (1.4)	18.9 (3.2)	20.6 (3.6)		RNAi x UVB§		

LMW phenolics, low-molecular weight phenolics; monogluc., monoglucoside; der., derivative; DPPG, dihydroxypropiophenone-3-glucoside; digluc., diglucoside; myr-3-gal, myricetin 3-galactoside; myr-3-gluc, myricetin 3-glucoside; myr-3-arab, myricetin 3-arabinoside; methylmyr-3-gluc; myricetin methyl ether 3-glucoside; quer-3-gal, quercetin 3-galactoside; quer-3-gluc, quercetin 3-glucoside; quer-3-arab, quercetin 3-arabinoside; quer-3-rham, quercetin 3-rhamnoside; kaemp-3-rhamn, kaempferol 3-rhamnoside; dicoum., dicoumaroyl; gallocat., gallocatechin; olig., oligomer; CT, condensed tannins.

–, not detected; ***, P<0.001; **, 0.001<P<0.01; *, 0.01<P<0.05

[†]Quantification in leaves of DFRi plants was interfered by an overlapping peak of ampelopsin.

[‡]Total concentration of stem LMW phenolics and stem phenolic acids excludes the protocatechuic acid, which was not quantified in all plants. For protocatechuic acid, means, standard errors and statistics are based on 48 plants without salicin added as internal standard.

[§]Interactive effects of RNAi and UVB were observed in the following leaf phenolics: **Under ambient UVB dose, total flavanones increased in DFRi and ANRi leaves compared to the control line (P<0.001 for both), and the enhanced UVB dose had an increasing effect on these compounds within DFRi leaves (P<0.001)**. Under ambient UVB dose, flavanone 1 increased in DFRi and ANRi leaves compared to the control line (P<0.001 for both), and the enhanced UVB dose had an increasing effect on this compound within DFRi leaves (P<0.001). Under ambient UVB dose, flavanone 2 increased in DFRi leaves compared to the control line (P=0.003), and the enhanced UVB dose had an increasing effect on this compound within DFRi leaves (P<0.001). Under ambient UVB dose, flavanone 3 increased in DFRi leaves compared to the control line (P=0.002), and the enhanced UVB dose had an increasing effect on this compound within DFRi leaves (P<0.001). **Under ambient UVB dose, total dihydroflavonols increased in DFRi leaves compared to the control line (P<0.001), and the enhanced UVB dose had an increasing effect on these compounds in DFRi leaves (P<0.001).** Under ambient UVB dose, dihydroflavonol 1 increased in DFRi leaves compared to the control line (P=0.009), and the enhanced UVB dose had an increasing effect on this compound in DFRi leaves (P<0.001). Under ambient UVB dose, diglucoside of ampelopsin was unchanged in DFRi, ANSi or ANRi leaves compared to the control line (P>0.05 for all comparisons), but the enhanced UVB dose had an increasing effect on this compound in DFRi leaves (P<0.001). Under ambient UVB dose, monoglucoside of ampelopsin increased in DFRi leaves compared to the control line (P<0.001), and the enhanced UVB dose had an increasing effect on this compound in DFRi leaves (P<0.001). Under ambient UVB dose, dihydroflavonol 2 increased in DFRi leaves (P=0.007), and the enhanced UVB dose had an increasing effect on this compound in DFRi leaves (P=0.002). Under ambient UVB dose, monoglucoside of taxifolin increased in DFRi leaves (P<0.001), and the enhanced UVB dose had an increasing effect on this compound in DFRi leaves (P<0.001). **Under ambient UVB dose, total quercetins increased in ANRi leaves compared to the control line (P<0.001), and the enhanced UVB dose had an increasing effect on these compounds within DFRi (P=0.004) and ANSi leaves (P=0.032).**

Interactive effects of RNAi and UVB were observed in the following stem phenolics: Under ambient UVB dose, dihydroflavonol 1 increased in DFRi stems compared to the control line (P<0.001), whereas the enhanced UVB dose had a decreasing effect on this compound within DFRi stems (P<0.001). Under ambient UVB dose, dihydroflavonol 2 increased in DFRi stems compared to the control line (P<0.001), and the enhanced UVB dose had an increasing effect on this compound within DFRi stems (P<0.001). **Under ambient UVB dose, total myricetins increased in DFRi and ANRi stems compared to the control line (P<0.001 for both), and the enhanced UVB dose had an increasing effect on these compounds within ANRi stems (P<0.001).** Under ambient UVB dose, myricetin 3-galactoside increased in DFRi and ANRi stems compared to the control line (P<0.001 for both), and the enhanced UVB dose had an increasing effect on myricetin 3-galactoside within ANRi stems (P<0.001). **Under ambient UVB dose, total condensed tannins decreased in DFRi, ANSi and ANRi stems compared to the control line (P<0.001, P<0.001 and P=0.011, respectively), and the enhanced UVB dose had a decreasing effect on these compounds in stems of the control line (P=0.015).** Under ambient UVB dose, insoluble condensed tannins ANSi stems decreased compared to the control line (P<0.007), and the enhanced UVB dose had a decreasing effect on these compounds in stems of the control line (P=0.012).

Methods S1. The coding sequences of putative *BpDFR1*, *BpANS*, and *BpANR* genes used for generating the transgenics. The sequence beginning the translation is underlined.

BpDFR1:

gcactagacatatttgcaacgttcgtaatttataataagcatggggtcggagggtgaaaccgtttgcgtcaccggcgcggctggttttatcggatc
atggctcgtcatgaggctcctagagcgcggttacgccgtccgagccaccgtgcgagacccagagaatatgaagaaggtaaagcatttgctaga
gctgccaaaggcgaagactcacttgactctgtggaaggctgacctggctgaggagggaagctttgatgaagcaatcaaaggttgcagcggggtt
ttccatgtcgccacacccatggatttcgaatccaaggaccctgagaatgaagtgataaagccaacgataaacgggttgttaggaatcatgaaag
cgtgtgtgaaagcaaaaactgttcgaagacttgtattcacatcctctgcaggaactgtcaatgttgaagagcaccaaaagccagtctacgatga
aacctgctggagtgatgttgaattttgcaggaccaaaaagatgactggatggatgtatttcgtctccaagacacttgctgagcaagccgcgtgga
agtttgccaaagaaaacggtttggattttataactgttataccacctcttgtggttggtccattcatcatgcaatcaatgccgcctagtcttattaca
gcactttcccctatcaccggaaatgaaggtcattactcgataattaagcaaggccaattcgtgcacttggatgacctctgcaatagtcacatatttt
tgtttgagcatcccaaatcggaggggaggtatatatgctccgccgccgatgccacaattcacgacattggaaaattgctaagagaaaaataccc
cgagtataatgttcctacaaagttcaaaggcatcgacgagaagttggagattgtgtctttctcttccaaaaaggttaaagaattgggatttgagtt
caaatacagcttggaggacatgtttgtaggagctgttgaaacatgccgatccaagggattgcttcctcctgccgccgttgaaaaccatgccaacg
gcaagaaccatgattgaaaatgtgtcggcatgttgcgtgacagggctttatgttggggaagtaaaaataatttttttttttttttaaaaaaaaatc
atctttcagcctagctatgaaatgaggggtatttgctaatggggctttctcgttttattcaaagttgttatggggattaatctgttgaaaaattttaaa
aaacttatctaaaaaagaatgatgatttggttcctaaaaaaaaaaaaaaaaaaaaaaaaaaaaaaaaaaaaaaaaaaaaaaaaaaaa
accccggggggggggccccgccccccattccccctttagggggggggtttaaaaattccgggggccggtttttaaaaagagggggg

BpANS:

atttacaaataaattaaaagaaagaaagaactttgtgaggatggtgacttcagttgcaccaagagttgagagcttgtcaagcagtgggatccaa
gcaatccccaaggagtacgtgaggccccaagaggagctaaacagcatcggcaacatcttcgacgaagagaaaaaggaagaagggcctcaag
ttccgaccattgacttgaaaaacatagagtccgaggatcaggctgtcagggaaaaatgccgggaggagttgaagagagctgccgtggaatggg
gtgtcatgcaccttgtgaaccacggcatctccgacgagctcctcgagcgggtgaagaaggccggaaaggtcttctttgatctgcccgtggaggag
aaggagaagtacgctaatgaccaggccttggggaagattcaaggctatggcagcaagctagcgaataacgctagcgggcagcttgaatggga
ggactatttcttccaccttgtttaccctgaggacaagcgtgacttgtccatctggcctaagacgcccactgactacattgtggcaacaaccgagta

cgcaaagcaactgagaatcctagcgagcaaggtattttctgtactatcccttggcctgggattggaagaagggaggctagagaaggaagtcgg

cggcctggaagaactacttctccaaatgaaaatcaattactacccgatctgccctcagccggagctggctctcggcgtcgaagctcacaccgacg

tgagcgccctcactttcattctccacaacatggtccccggcctgcaactcttctacgagggcaaatgggtgacggcgaaatgtgttcccaactcca

taatcatgcacatcggcgacaccttagagatcctcagcaaccggaagtacaagagtattcttcacagggggctggtgaacaaggagaaggtta

ggatctcgtgggcggttttctgtgagccgccgaaggagaagattattctgaagcctctgccggaggttgtatcggaggaagagccggcgatcttc

ccaccgaggacctttgctcagcatattcagcacaagctgttcaggaagacccaggatgctcttgacgctaagtaatgaattgtcatctttgttgttg

atacgtcgtttcatggctctgtcatgtcgtttcaatatgtattggtttattgaggcttttattgtggcaggtataggccattaggttatgggtttgtcct

ttgctattggctagtacaccatgtctactttttaaataagaataatattcatgtgttatatgattgaaaaaaaaaaacatagtcaagtatttttactt

ttataagagattttgtttgggatttagcgattcgaaaaaaaaaaaaaaaaaaaaaactcgaggggggg

BpANR:

attaccttccccgctgcctctgctttgaccctcaaatattgctttcgactttcgatcattactttttttgtggaaaaatcagagagagtgagtgagagt

tagcatcaaaattaaagtccatggctactgatcagcatatcggaaagaagactgcctgcgtcgtcggcggcaccgggttcgtcgcatctttgctg

gtcaagctcttgctggagaaggactatgccgtcaacaccaccgtcagggaccctgaaaatgagaagaagatctctcacctcagagcactacaa

gatttgggggacctaaaaatctttggagcagatctaaccgatgaaggaagctttgacgctcctatagcaggttgtgaacttgtgttccatcttgca

acacctgttaactttgcttcagaagatccagagaatgacatgatcaagccagcaatccaaggagtacataatgttctgaaagcatctgcaagag

caaagactgttaaacgtgtcgttttgacatcatctgcagctgctgtaacgatcaacaagctcaatgggactggtttagtcatggacgaaagccact

ggaccgacgtcgagtttttgagtactgagaagccacctacttggggttatcctgcttccaagacactggctgagaaagcagcttggaaattcgct

gaagaaaacaatattgatctcatcacagtgattcctactcttatggctggtgcttctctcactccggacgtccccagcagtgtttgtctagctatga

gtctaataactggcaatgaattcctcataaatggaatgaaaggtatgcaaatgctgtcaggctcaatatctatcacacatgtggaggatgtttgcc

gggcccaaatatttttggctgagaaagaatctgcttctggtcgatatatatgctgtggtgtcaataccagtgttcctgagcttgcaaagttcctcaa

taacagataccctaactacaaagtcccaactgatttcggagattttccctccaaggccaagttggccttatcttcagagaagcttatcaaagaag

ggttcagctttaagtacgggattgaagatatttatgaccaaaccgtggagtatttcaaagcaaagggcatgctgcagaactaagacaatggtttt

ttgttcctcttcaaagtgatgctctagtttttttttaattcatctatgctctaagcttggttgtatgcagtattgtataataacatctaaatttgcttga

ctttgatatgatagacttgtcgtttatcaagaatctaaagcaatctagcaactctgcttgaaactatcaagaatctaaagcaagataatgttatgc

atta

Methods S2. Identification of leaf LMW phenolics not observed in Thitz et al. (2020).

In this experiment, five new leaf compounds were identified based on UHPLC-Q-TOF/MS, or, when this was unavailable, based on UV-spectra and retention times in HPLC-UV-DAD chromatograms. Retention times (Rt) below refer to HPLC-UV-DAD samples in this experiment.

In addition to foliar gallocatechin identified and quantified according to Thitz et al. (2020), an additional gallocatechin derivative (Rt 4.5 min) with similar UV-spectrum was found. Thus, 'gallocatechin' of Thitz et al. (2020) was renamed as gallocatechin derivative 1, and the new compound as gallocatechin derivative 2. Proanthocyanidin oligomer (Rt 11.1 min) has been observed earlier from the same plant lines (Kosonen et al. 2015), and was quantified on 220 nm using catechin as a standard. Quercetin derivative 1 (Rt 24.5 min) was identified with HPLC-UV-DAD and quantified on 320 nm, using quercetin 3-galactoside as a standard. Dicoumaroyl astragalin (Rt 44.5 min) and kaempferol derivative 1 (Rt 45.2 min) were identified with HPLC-UV-DAD and quantified on 320 nm, using kaempferol 3-glucoside as a standard.

Methods S3. Flavan-3-ol subunits of condensed tannins identified from thiolysis samples.

The following terminal and extender flavan-3-ol subunits of condensed tannins were identified based on their UV-spectra and comparison of retention times to library samples from *Lespedeza stuevei* Nutt. and *Neptunia lutea* (Leavenw.) Benth, with well-characterized and structurally diverse condensed tannins (Naumann et al. 2018; https://doi.org/10.3390/molecules23092123): gallocatechin (GC; terminal Rt 5.0 min, extender Rt 20.7 min), epigallocatechin (EGC; terminal Rt 5.3 min, extender Rt 21.2 min), galloylated gallocatechin (GCg; not observed among terminal subunits, extender Rt 24.3 min), catechin (CAT; terminal Rt 6.9 min, extender Rt 26.1 min), epicatechin (EC; terminal Rt 8.4 min, extender Rt 28.1 min), galloylated catechin (Cg; not observed among terminal subunits, extender Rt

35.6 min) and galloylated epicatechin (ECg; not observed among terminal subunits, extender Rt 35.7 min), and quantified based on their peak area.

Free flavan-3-ols (GC, EGC, C, EC) were identified from unreacted plant extracts similarly to terminal flavan-3-ols observed in the thiolysed samples, using a program modified from Scioneaux et al. (2011), where the following linear gradient for 0.1% TFA in acetonitrile (v/v) was used: 2% (0–4 min), 2–34% (4–35 min), 34–80% (35–37 min), followed by re-equilibration). After establishing the retention times of free flavan-3-ols in this program, they were identified as GC (Rt 15.3 min), EGC (Rt 19.5 min), CAT (Rt 20.7 min) and EC (Rt 23.5 min) and quantified based on their peak area.

Condensed tannins (proanthocyanidins) comprise (epi)-catechin subunits and their 3'-O-gallate esters and/or (epi)-gallocatechin subunits and their esters. The (epi)-catechin subunits and their esters yield the anthocyanidin upon cleavage in anhydrous acidic alcohol. The (epi)-gallocatechin subunits and their esters yield delphinidin.

Methods S4. Initial and final fixed effect structures of models on stem height and diameter (L and D; 8 repeated measurements), leaf chlorophyll index (chl; 6 repeated measurements), variable chlorophyll fluorescence (Fv/Fm; 2 repeated measurements) and gas exchange (A_n and E; 2 repeated measurements). All models included random intercepts for individual plants, nested within lines.

Fixed terms *RNAi* (with three levels: DFRi, ANSi, or ANRi) specify the enzyme inhibited with RNA interference; *UVB* refers to enhanced UVB treatment; *L0* and *D0* refer to the initial height and diameters measured from the same plant individuals five days before the onset of experiment; *chl1* refers to the initial chlorophyll content index measured on the first repeated measurement (June 22, 2016), *week* and $week^2$ specify the time of repeated measurement as weeks since the onset of experiment and its square; and *time* and $time^2$ specify the time of measurement as hours since midnight and its square. Terms specifying interactions between main effect terms *A* and *B* are marked as *A:B*. Conditional F-tests were done on nested models to test whether the main or interactive terms

(excluding *intercept*, *RNAi* and *UVB*) improved model fit, and interactive effects were left out when not significant at $P<0.05$.

Initial model structures:

sqrt(L): intercept + RNAi + UVB + L0 + week + $week^2$ + RNAi:UVB + RNAi:week + UVB:week + RNAi:UVB:week + RNAi:$week^2$ + UVB:$week^2$ + RNAi:UVB:$week^2$

D: intercept + RNAi + UVB + D0 + week + $week^2$ + RNAi:UVB + RNAi:week + UVB:week + RNAi:UVB:week + RNAi:$week^2$ + UVB:$week^2$ + RNAi:UVB:$week^2$

chl: intercept + RNAi + UVB + chl1 + week + $week^2$ + RNAi:UVB + RNAi:week + UVB:week + RNAi:UVB:week + RNAi:$week^2$ + UVB:$week^2$ + RNAi:UVB:$week^2$

Fv/Fm: intercept + RNAi + UVB + week + time + $time^2$ + RNAi:UVB

sqrt(E): intercept + RNAi + UVB + week + time + $time^2$ + RNAi:UVB

A_n: intercept + RNAi + UVB + week + time + $time^2$ + RNAi:UVB

WUE: intercept + RNAi + UVB + week + time + $time^2$ + RNAi:UVB

Final model structures:

sqrt(L): intercept + RNAi + UVB + L0 + week + $week^2$ + RNAi:week + RNAi:$week^2$

D: intercept + RNAi + UVB + D0 + week + $week^2$ + RNAi:week + UVB:week + RNAi:$week^2$ + UVB:$week^2$

chl: intercept + RNAi + UVB + chl1 + week + $week^2$ + RNAi:week + RNAi:$week^2$

Fv/Fm: intercept + RNAi + UVB + week + time + $time^2$

sqrt(E): intercept + RNAi + UVB + week + time + $time^2$ + RNAi:UVB

A_n: intercept + RNAi + UVB + week + time + $time^2$

WUE: intercept + RNAi + UVB + week + time + $time^2$
